# Transcriptomic analysis of Pak Choi under acute ozone exposure revealed regulatory mechanism against ozone stress

**DOI:** 10.1186/s12870-017-1202-4

**Published:** 2017-12-08

**Authors:** Lu Zhang, Bin Xu, Tao Wu, Mu-xuan Wen, Lian-xue Fan, Zhao-zhong Feng, Elena Paoletti

**Affiliations:** 1 College of Horticulture and Landscape Architecture, Northeast Agricultural University, Harbin, China; 2 College of Agro-grassland Science, Nanjing Agricultural University, Nanjing, China; 3 State Key Laboratory of Urban and Regional Ecology, Research Center for Eco-Environmental Sciences, Chinese Academy of Sciences, Beijing, China; 4 Institute of Sustainable Plant Protection, National Research Council, Florence, Italy

**Keywords:** Glutathione metabolism, Ozone, Pak Choi, RNA-Seq, Transcriptome

## Abstract

**Background:**

Ground-level ozone (O_3_) is one of the major air pollutants, which cause oxidative injury to plants. The physiological and biochemical mechanisms underlying the responses of plants to O_3_ stress have been well investigated. However, there are limited reports about the molecular basis of plant responses to O_3_. In this study, a comparative transcriptomic analysis of Pak Choi (*Brassica campestris ssp. chinensis*) exposed to different O_3_ concentrations was conducted for the first time.

**Results:**

Seedlings of Pak Choi with five leaves were exposed to non-filtered air (NF, 31 ppb) or elevated O_3_ (E-O_3_, 252 ppb) for 2 days (8 h per day, from 9:00–17:00). Compared with plants in the NF, a total of 675 differentially expressed genes (DEGs) were identified in plants under E-O_3_, including 219 DEGs with decreased expressions and 456 DEGs with increased expressions. Kyoto Encyclopedia of Genes and Genomes (KEGG) analyses revealed that O_3_ stress invoked multiple cellular defense pathways to mitigate the impaired cellular integrity and metabolism, including ‘glutathione metabolism’, ‘phenylpropanoid biosynthesis’, ‘sulfur metabolism’, ‘glucosinolate biosynthesis’, ‘cutin, suberine and wax biosynthesis’ and others. Transcription factors potentially involved in this cellular regulation were also found, such as AP2-ERF, WRKY, JAZ, MYB etc. Based on the RNA-Seq data and previous studies, a working model was proposed integrating O_3_ caused reactive oxygen burst, oxidation-reduction regulation, jasmonic acid and downstream functional genes for the regulation of cellular homeostasis after acute O_3_ stress.

**Conclusion:**

The present results provide a valuable insight into the molecular responses of Pak Choi to acute O_3_ stress and the specific DEGs revealed in this study could be used for further functional identification of key allelic genes determining the O_3_ sensitivity of Pak Choi.

**Electronic supplementary material:**

The online version of this article (10.1186/s12870-017-1202-4) contains supplementary material, which is available to authorized users.

## Background

Tropospheric ozone (O_3_) is generated by reactions between reactive nitrogen oxides (NO_x_) and volatile organic compounds (VOCs) in the presence of sunlight, and is one of the key atmospheric pollutants due to anthropogenic activities [1]. Ozone impairs the health of human beings, and also causes serious threats to crops, forests and other ecosystems [2, 3].

At the cellular level, we know now a great deal about the mechanisms by which O_3_ causes oxidative damages to plants [4, 5]. Ozone enters the leaves through the stomata and dissolves in apoplastic solutes, where it then subsequently triggers the production of reactive oxygen species [ROS, such as superoxide anion radical (O_2_^.-^) and hydrogen peroxide (H_2_O_2_)] in the plant cell wall in a highly regulated manner, and ultimately leads to activation of defense and other metabolic signaling pathways among which ROS scavenging is the most remarkable cellular response to high O_3_ concentration [6–8].

Analyses on transcriptomic profiling in response to O_3_ stress have been reported in a number of model plants, crops and trees, such as Arabidopsis [9–11], *Medicago truncatula* [12], pepper [13], soybean [14], and aspen tree [15], by means of microarray or next-generation sequencing technologies. Many kinds of leafy vegetables are sensitive to O_3_ pollution [16]. However, knowledge about transcriptomic response to O_3_ stress in leafy vegetables is very limited [17].

Pak Choi (*Brassica campestris* L. ssp. *chinensis* L. Makino) is one of the most consumed vegetables in East Asia with high healthy and commercial values [18]. In our previous study, we found that Pak Choi is sensitive to acute O_3_ fumigation as reflected by decreased chlorophyll content, increased anthocyanin content, damaged cell membrane integrity, enhanced antioxidative enzyme activities, depressed photosynthetic rate and stomatal conductance, inhibited maximal quantum yield and effective quantum yield of PSII photochemistry [19]. However, the underlying molecular mechanisms for such physiological alterations are not clear. To our best knowledge, there is no previous report on transcriptomic changes of Pak Choi upon O_3_ exposure. The goal of this study was to identify and characterize key genes encoding the protein and metabolic pathways involved in O_3_ responses in Pak Choi by using RNA-Seq as the methodological approach with *Brassica rapa subsp. pekinensis* (Lour.) Hanelt as the reference genome.

## Results

### RNA-sequencing of Pak Choi and DEGs between different O_3_ treatments

The second fully expanded leaves from the top of Pak Choi plants under NF and E-O_3_ were used for RNA-Seq. The sequencing results were deposited in the NCBI SRA database (Accession number: SRP100739). In total, 172,380,454 and 164,874,922 raw reads were generated from the three replicated NF libraries and the three replicated E-O_3_ libraries, respectively (Additional file 1: Table S1). To ensure the quality of the libraries, adaptor reads, ambiguous reads and low-quality reads were removed (Additional file 2: Figure S1). Finally, a total of 165,289,336 and 158,294,170 clean reads were obtained for NF and E-O_3_, respectively (Additional file 1: Table S1), among which 67.63% reads in NF and 69.89% reads in E-O_3_ were mapped in the *Brassica* database (BRAD) (Additional file 1: Table S1). The principle component analysis (PCA) of the RNA-seq data showed that reads of the three NF libraries clustered together while those of E-O_3_ clustered together (Additional file 3: Figure S2), further supporting the validity of the experimental design and RNA-seq data. Comparing Pak Choi plants under E-O_3_ exposure with those in the NF, a total of 675 DEGs were identified, including 219 DEGs with decreased expressions and 456 DEGs with increased expressions (Additional file 4).

### Verification of RNA-Seq data by qRT-PCR

Transcriptional levels of 12 selected DEGs were examined by qRT-PCR in order to validate the reliability of the RNA-Seq data. Among the selected DEGs, three encoded proteins with oxidoreductase activities, two involved in glutathione metabolism, one involved in cell wall formation, and the rest encoded stress-related transcription factors (Table 1). The result showed that qRT-PCR data were in similar trend to those of the RNA-Seq, proving the reliability of RNA-Seq results (Table 1).

**Table 1:** The expression patterns of selected genes in plants under non-filtered air (NF) or elevated O_3_ (E-O_3_) using real-time quantitative RT-PCR and RNA-Seq

Gene	Relative expression of the target gene by qRT-PCR		FPKM value from RNA-Seq		Gene annotation
	NF	E-O_3_	NF	E-O_3_	
Bra003517	1.07 ± 0.08 b	2.20 ± 0.26 a	0	7.74	glutaredoxin family protein
Bra013923	1.12 ± 0.32 b	85.57 ± 9.21 a	0	5.05	XTR9 (XYLOGLUCAN ENDOTRANSGLYCOSYLASE 9); hydrolase, acting on glycosyl bonds/ hydrolase, hydrolyzing O-glycosyl compounds/ xyloglucan:xyloglucosyl transferase
Bra020878	1.54 ± 0.58 b	93.07 ± 10.02 a	0	5.90	basic helix-loop-helix (bHLH) family protein
Bra028899	1.08 ± 0.07 b	16.48 ± 4.00 a	0	12.79	transcription factor
Bra031485	0.98 ± 0.12b	26.54 ± 2.01 a	20.40	1091.02	oxidoreductase
Bra038089	0.70 ± 0.26 b	743.15 ± 58.96 a	3.07	329.91	lipid binding/ structural constituent of cell wall
Bra034061	0.94 ± 0.13 b	32.32 ± 5.79 a	58.71	1332.50	ATGSTU8 (GLUTATHIONE S-TRANSFERASE TAU 8); glutathione transferase
Bra035732	2.23 ± 1.16 b	9.17 ± 2.00 a	5.95	85.43	AP2 domain-containing transcription factor, putative
Bra010802	1.16 ± 0.15 b	4.74 ± 0.94 a	2.48	26.20	ATGA2OX2 (GIBBERELLIN 2-OXIDASE); gibberellin 2-beta-dioxygenase
Bra012938	1.47 ± 0.58 b	6.45 ± 0.66 a	30.81	318.51	ERF104; ethylene-responsive element-binding family protein
Bra009445	0.78 ± 0.33 b	100.98 ± 38.70a	0	69.96	KCS19 (3-KETOACYL-COA SYNTHASE 19); acyltransferase/ catalytic/ transferase, transferring acyl groups other than amino-acyl groups
Bra025833	0.95 ± 0.07 a	0.30 ± 0.03 b	5.92	0	CAT1 (CATALASE 1); catalase

The relative quantitation of gene expression was conducted via the 2^−ΔΔCt^ method, with actin as an endogenous reference. Data from three biological replicates were used to calculate the mean and standard deviation in DPS based on Student’s t-test. Values followed by different letters indicate significant difference at *P* < 0.05. FPKM: the expected number of Fragments *per* Kilobase of transcript sequence *per* Millions base pairs sequenced

### Functional classification of DEGs

**Fig. 1:**
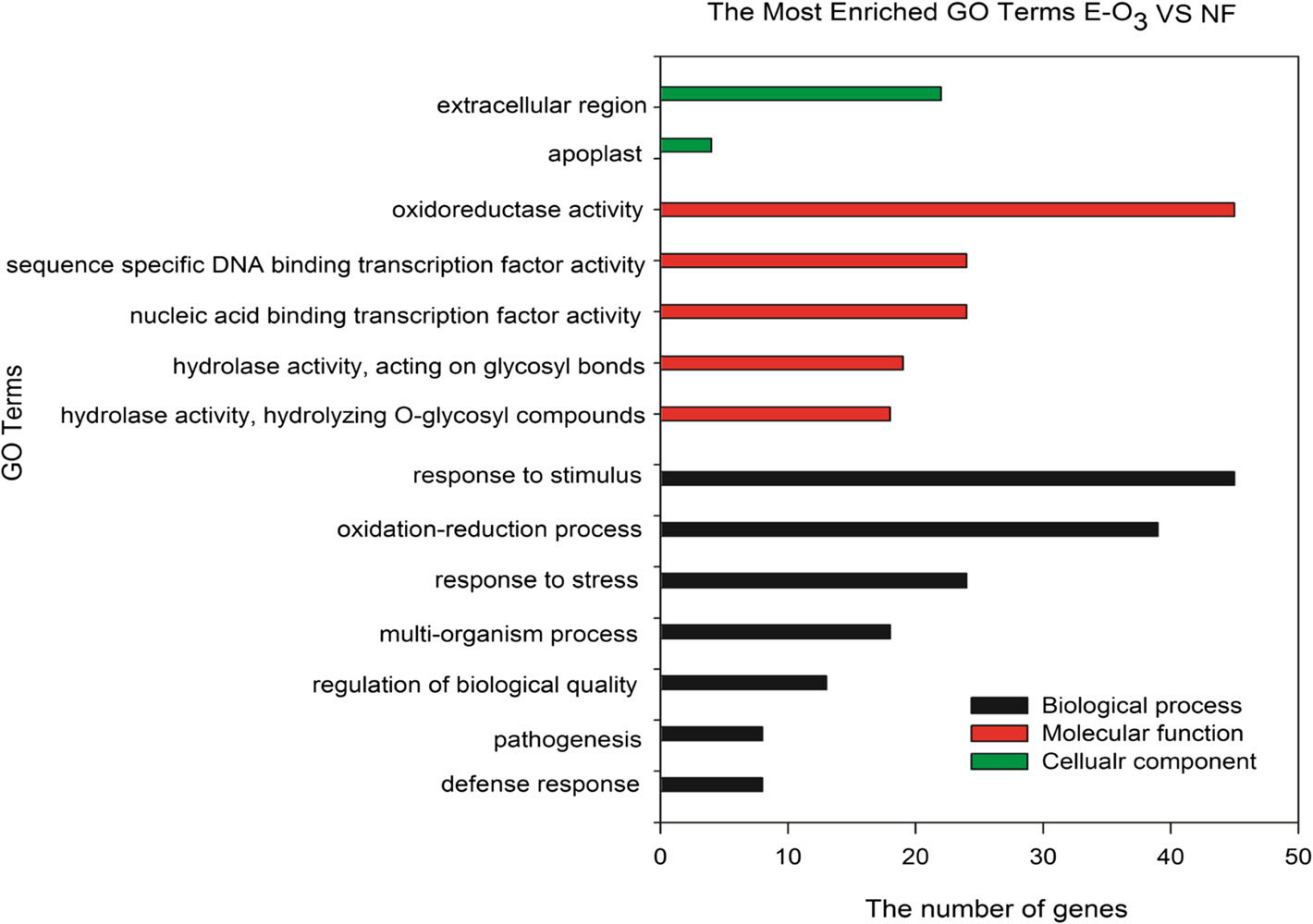
Gene Ontology (GO) enrichment of differentially expressed genes (DEGs) with increased expressions in leaves of Pak Choi under elevated O_3_ (E-O_3_) compared with non-filtered air (NF)

**Fig. 2:**
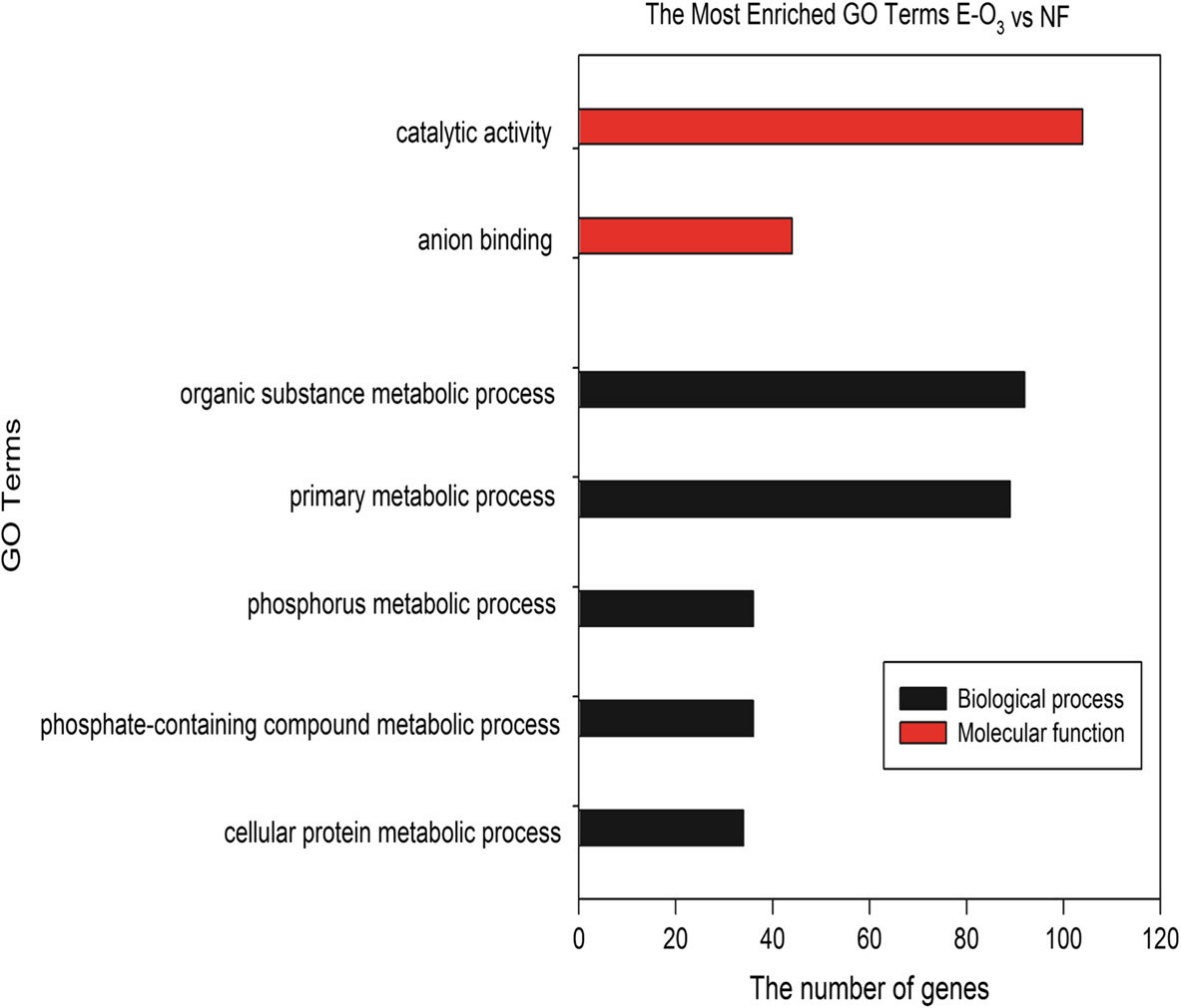
Gene Ontology (GO) enrichment of differentially expressed genes (DEGs) with decreased expressions in leaves of Pak Choi under elevated O_3_ (E-O_3_) compared with non-filtered air (NF)

GO enrichment analysis were conducted in order to classify the possible functions of DEGs. From the DEGs with increased expressions in plants under E-O_3_ exposure, the top enriched terms were ‘response to stimulus’, ‘oxidation-reduction process’, ‘response to stress’, ‘multi-organism process’, ‘regulation of biological quality’, ‘pathogenesis’, and ‘defense response’ in the category of biological process; in the molecular function category, the ‘oxidoreductase activity’, ‘sequence specific DNA binding transcription factor activity’, ‘nucleic acid binding transcription factor activity’, ‘hydrolase activity, acting on glycosyl bonds’ and ‘hydrolase activity, hydrolyzing O-glycosyl compounds’ were the mostly highly enriched; ‘extracellular region’ and ‘apoplast’ were highly enriched in the category of cellular component (Fig. 1). For the DEGs with decreased expressions in plants upon E-O_3_ treatment, ‘organic substance metabolic process’, ‘primary metabolic process’, ‘phosphorus metabolic process’, ‘phosphate-containing compound metabolic process’ and ‘cellular protein metabolic process’ were the highly enriched ones in the category of biological process; and in the category of molecular function, the top enriched terms were ‘catalytic activity’ and ‘anion binding’ (Fig. 2).

**Fig. 3:**
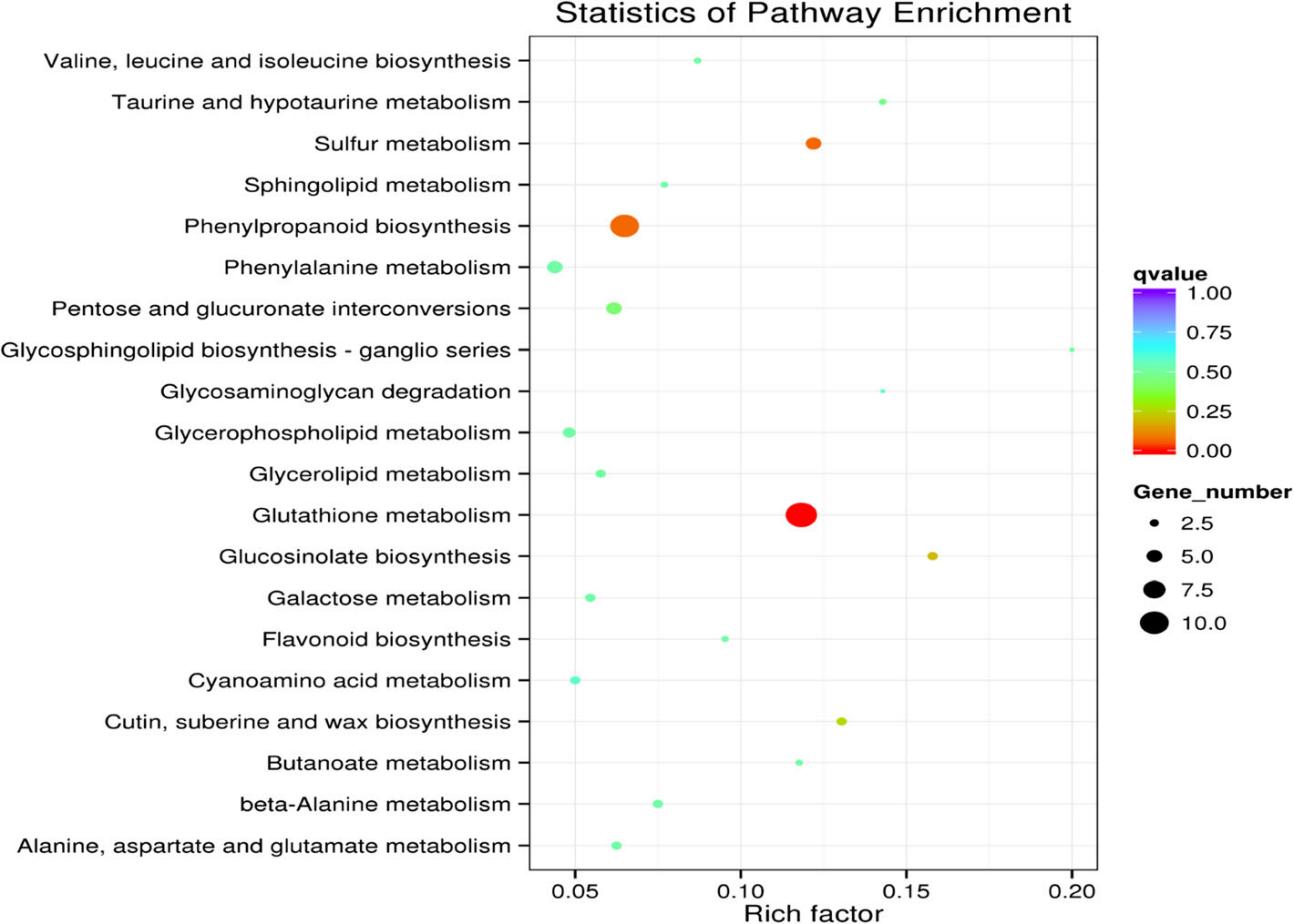
Kyoto Encyclopedia of Genes and Genomes (KEGG) enrichment of differentially expressed genes (DEGs) with increased expressions in leaves of Pak Choi under elevated O_3_ (E-O_3_) compared with non-filtered air (NF)

**Fig. 4:**
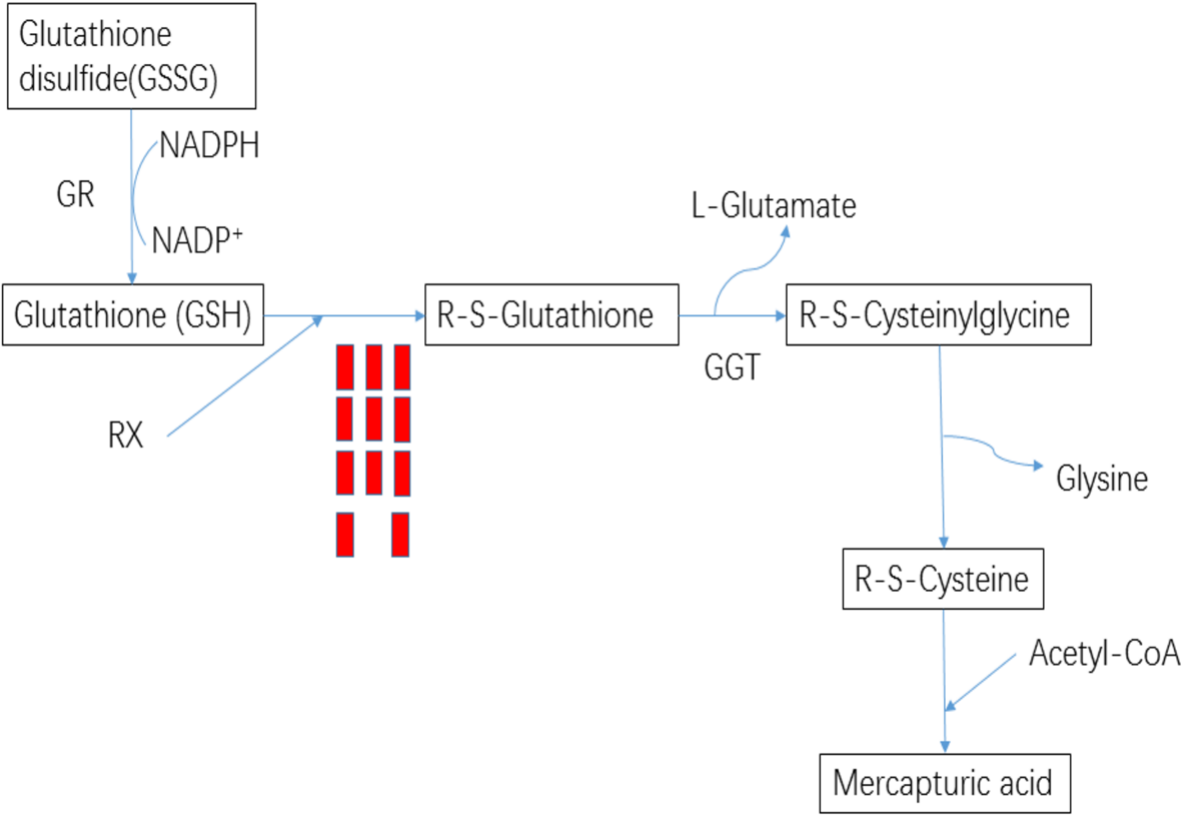
Differential expressed genes predicted to be involved in the ‘glutathione metabolism’ pathway. Each red block means one DEG which had increased expression

**Fig. 5:**
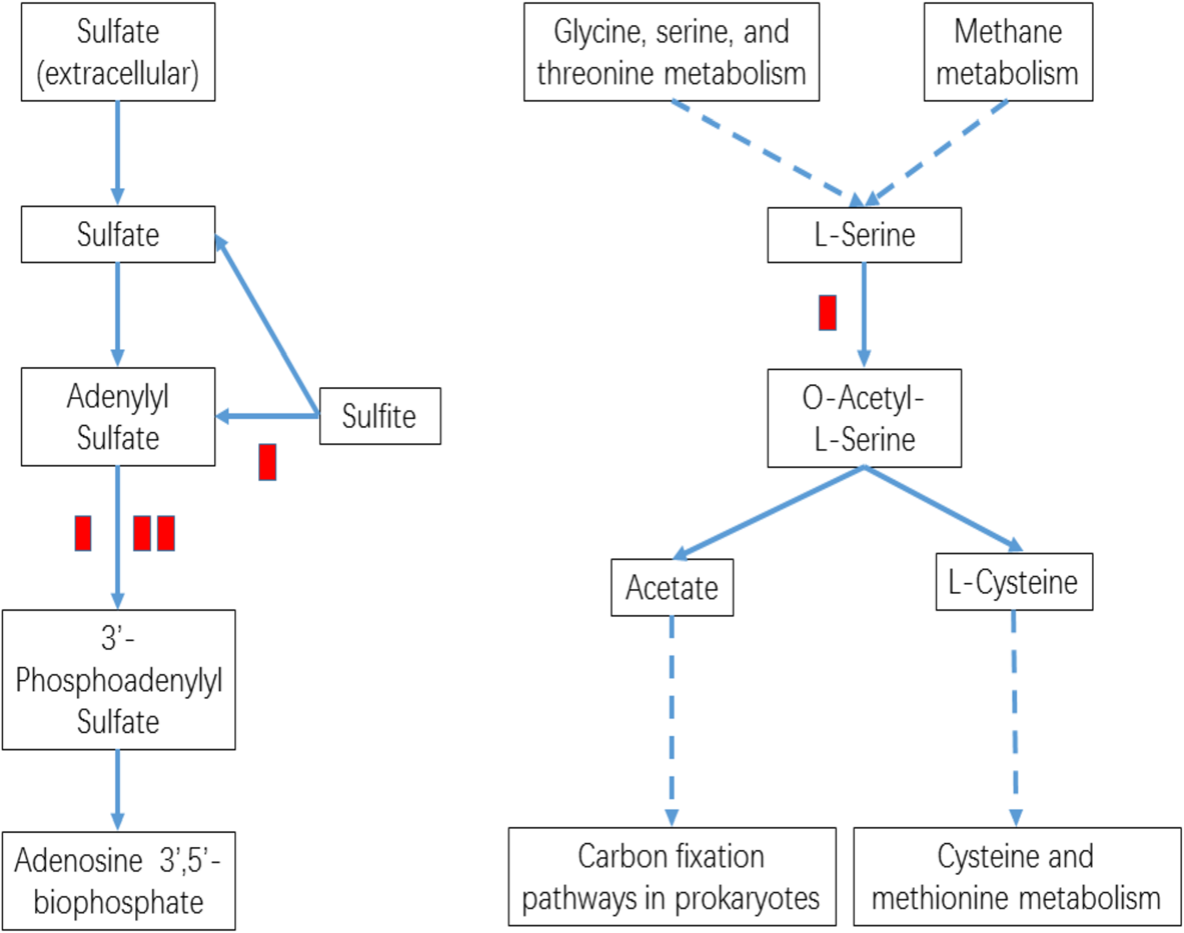
Differential expressed genes predicted to be involved in the ‘sulfur metabolism’ pathway. Each red block means one DEG which had increased expression

**Fig. 6:**
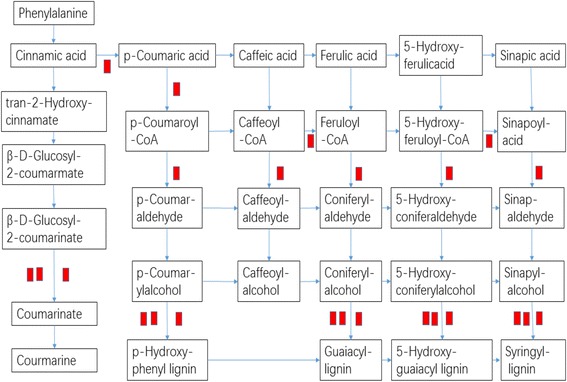
Differential expressed genes predicted to be involved in the ‘phenylpropanoid biosynthesis’ pathway. Each red block means one DEG which had increased expression-

**Fig. 7:**
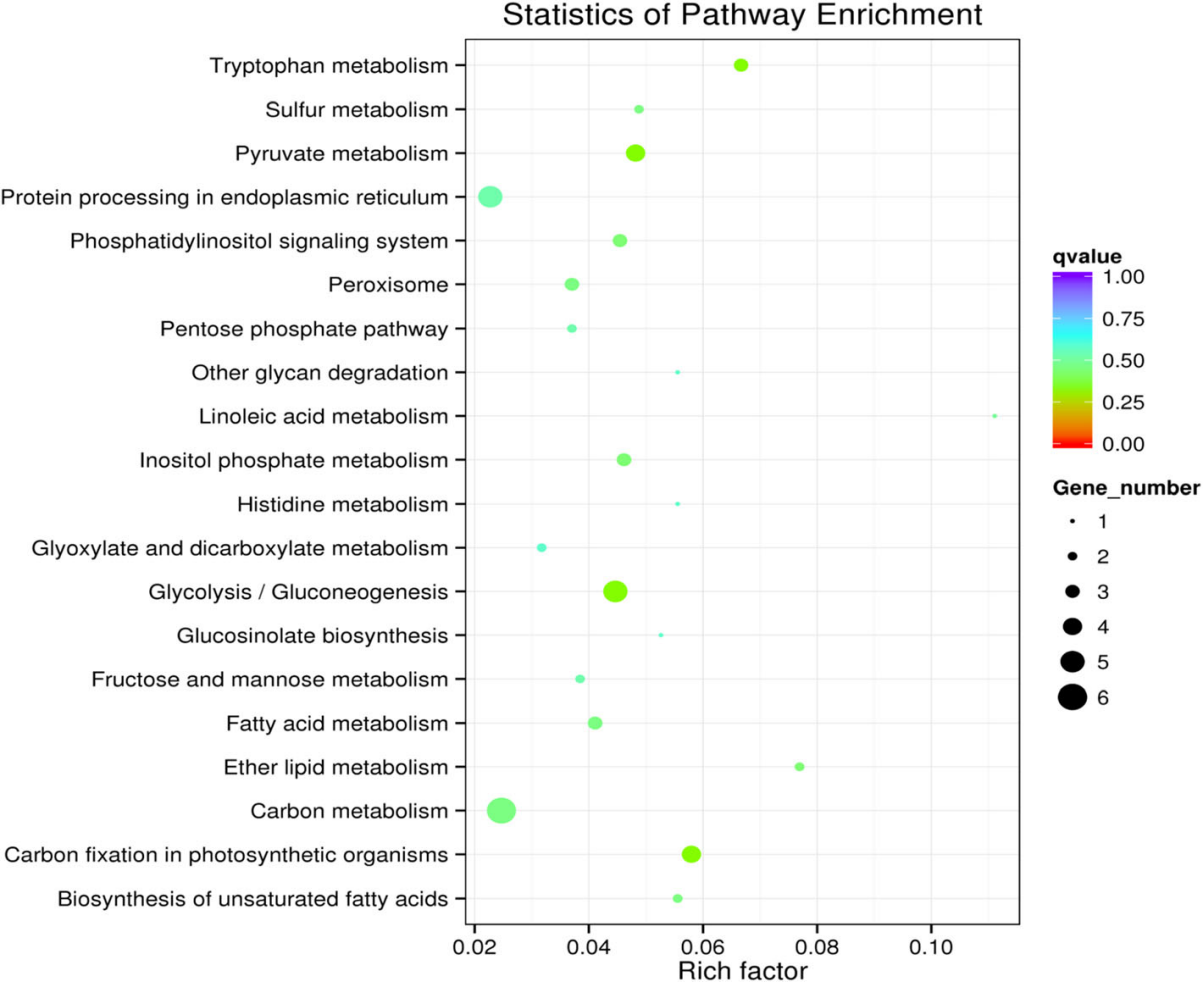
Kyoto Encyclopedia of Genes and Genomes (KEGG) enrichment of differentially expressed genes (DEGs) with decreased expressions in leaves of Pak Choi under elevated O_3_ (E-O_3_) compared with non-filtered air (NF)

**Fig. 8:**
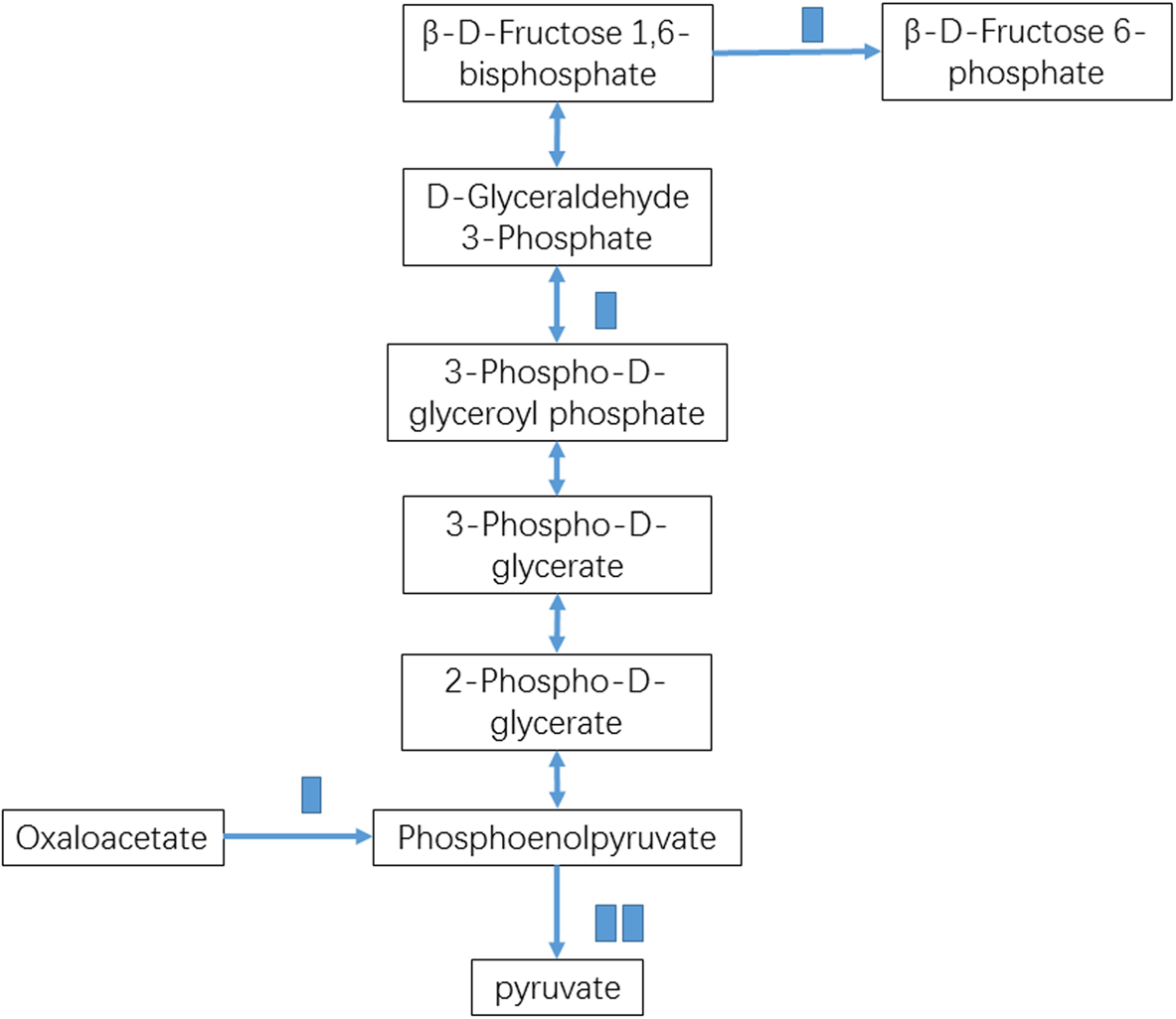
Differential expressed genes predicted to be involved in ‘glycolysis/gluconeogenesis’ pathway. Each blue block means one DEG which had decreased expression

KEGG pathway enrichment analysis was performed to reveal the enriched pathways. As shown in Fig. 3, enriched pathways of DEGs with increased expressions in plants under E-O_3_ were ‘glutathione metabolism’ [KEGGmap:ath00480], ‘phenylpropanoid biosynthesis’ [KEGGmap:ath00940], ‘sulfur metabolism’ [KEGGmap:ath00920], ‘glucosinolate biosynthesis’ [KEGGmap:ath00966], ‘cutin, suberine and wax biosynthesis’ [KEGGmap:ath00073], ‘pentose and glucoronate interconversions’ [KEGGmap:ath00040] and ‘taurine and hypotaurine metabolism’ [KEGGmap:ath00430]. Eleven DEGs between different O_3_ treatments were involved in ‘glutathione metabolism’ (Table 2), ten were in ‘phenylpropanoid biosynthesis’, and five were in ‘sulfur metabolism’ (Figs. 4, 5, and 6). The enriched pathway of DEGs with decreased expressions in plants under E-O_3_ were ‘carbon fixation in photosynthetic organisms’ [KEGGmap:ath00710], ‘glycolysis/gluconeogenesis’ [KEGGmap:ath00010], ‘tryptophan metabolism’ [KEGGmap:ath00380], ‘pyruvate metabolism’ [KEGGmap:ath00620], and ‘ether lipid metabolism’ [KEGGmap:ath00565] (Fig. 7). Four DEGs were involved in ‘carbon fixation in photosynthetic organisms’, five were in ‘glycolysis/gluconeogenesis’ (Fig. 8), three were in ‘tryptophan metabolism’, four were in ‘pyruvate metabolism’, and two were in ‘ether lipid metabolism’. Among them, some DEGs were involved in more than one metabolism pathway such as *Bra017856*, *Bra026068*, *Bra005526*, *Bra002822*, and *Bra009352*.

**Table 2:** The expression patterns of related genes in the glutathione metabolism pathway under non-filtered air (NF) or elevated O_3_ (E-O_3_)

		FPKM	
Gene ID	Gene annotation	NF	E-O_3_
Bra016250	ATGSTU11 (GLUTATHIONE S-TRANSFERASE TAU 11); glutathione transferase	192.79	640.92
Bra035029	ATGSTU22 (GLUTATHIONE S-TRANSFERASE TAU 22); glutathione transferase	0	31.89
Bra034061	ATGSTU8 (GLUTATHIONE S-TRANSFERASE TAU 8); glutathione transferase	58.71	1332.50
Bra039980	ATGSTU7 (*Arabidopsis thaliana* GLUTATHIONE S-TRANSFERASE TAU 7); glutathione transferase	365.32	1748.65
Bra025995	ATGSTU24 (GLUTATHIONE S-TRANSFERASE TAU 24); glutathione binding / glutathione transferase	59.65	1198.93
Bra025994	ATGSTU25 (GLUTATHIONE S-TRANSFERASE TAU 25); glutathione transferase	279.33	975.00
Bra039984	ATGSTU2 (ARABIDOPSIS THALIANA GLUTATHIONE S-TRANSFERASE TAU 2); glutathione transferase	1.18	21.17
Bra039982	ATGSTU4 (ARABIDOPSIS THALIANA GLUTATHIONE S-TRANSFERASE TAU 4); glutathione transferase	233.03	905.74
Bra026684	ATGSTU25 (GLUTATHIONE S-TRANSFERASE TAU 25); glutathione transferase	3.72	54.47
Bra026681	ATGSTU25 (GLUTATHIONE S-TRANSFERASE TAU 25); glutathione transferase	2.50	50.34
Bra032010	ATGSTF11 (GLUTATHIONE S-TRANSFERASE F11); glutathione transferase	305.56	564.83

*FPKM* the expected number of Fragments *per* Kilobase of transcript sequence *per* Millions base pairs sequenced

Transcription factors (TFs) play key roles in the regulation of gene expression. Candidate *TF* genes potentially involved in plant’s responses to O_3_ stress were further analyzed (Table 3). The results showed that most of the DEGs encoding transcription factors that positively associated with stress-tolerance (e.g. AP2-EREBP, WRKY, Trihelix, MYB, C2H2, TAZ, LOB, SNF2 and PHD) had increased expressions in plants under E-O_3_; while there were only three *TF* genes with decreased expressions encoding an Orphans protein (ARR15, negative regulator in cytokinin pathway), a bHLH family protein, and a Tify family protein under E-O_3_ (Table 3).

**Table 3:** Selected genes of transcription factors with changed expressions under non-filtered air (NF) or elevated O_3_ (E-O_3_)

			FPKM	
Group	Gene ID	Gene Annotation	E-O_3_	NF
bHLH	Bra000291	DNA binding/ transcription factor	3.40	19.54
	Bra011237	basic helix-loop-helix (bHLH) family protein	109.50	17.89
Trihelix	Bra005127	Trihelix transcription factor GT-3b	59.24	7.74
	Bra005688	Trihelix transcription factor GT-3a	85.79	5.53
	Bra028899	transcription factor	12.79	0
WRKY	Bra004370	WRKY57 transcription factor	18.79	4.263
	Bra036138	WRKY48 transcription factor	71.07	3.24
	Bra040926	WRKY28 transcription factor	86.83	3.15
MYB	Bra014929	Myb-related protein Myb4	17.72	2.186
AP2-ERF	Bra012938	Ethylene-responsive transcription factor ERF104	318.51	30.81
	Bra015660	Ethylene-responsive transcription factor ERF013	25.10	7.60
	Bra019777	DDF1 (DWARF AND DELAYED FLOWERING 1); DNA binding/ sequence-specific DNA binding/ transcription factor	37.12	2.12
	Bra024953	ERF5 (ETHYLENE RESPONSIVE ELEMENT BINDING FACTOR 5); DNA binding/ transcription activator/ transcription factor	203.15	28.95
	Bra026963	DDF1 (DWARF AND DELAYED FLOWERING 1); DNA binding/ sequence-specific DNA binding/ transcription factor	66.03	6.78
	Bra028290	CBF4 (C- REPEAT-BINDING FACTOR 4); DNA binding/ transcription activator/ transcription factor	74.88	1.76
	Bra035732	AP2 domain-containing transcription factor, putative	85.43	5.95
	Bra040158	ATERF6 (ETHYLENE RESPONSIVE ELEMENT BINDING FACTOR 6); DNA binding/ transcription factor	157.31	22.51
C2H2	Bra019477	zinc finger (C2H2 type) family protein	24.60	4.61
	Bra001752	AZF2 (ARABIDOPSIS ZINC-FINGER PROTEIN 2); DNA binding/ nucleic acid binding/ transcription factor/ transcription repressor/ zinc ion binding	152.39	18.96
	Bra020284	RHL41 (RESPONSIVE TO HIGH LIGHT 41); nucleic acid binding/ transcription factor/ zinc ion binding	22.37	4.41
Orphans	Bra015885	ARR15 (RESPONSE REGULATOR 15); negative regulator in the cytokinin-mediated signal transduction in Arabidopsis	3.05	14.43
Tify	Bra021923	JAZ7 (JASMONATE-ZIM-DOMAIN PROTEIN 7)	0	8.41
	Bra025713	JAZ1 (JASMONATE-ZIM-DOMAIN PROTEIN 1); protein binding	77.83	24.39
TAZ	Bra017839	BT5 (BTB AND TAZ DOMAIN PROTEIN 5); protein binding/ transcription regulator	24.37	4.69
LOB	Bra011942	LBD11 (LOB DOMAIN-CONTAINING PROTEIN 11)	41.51	4.21
SNF2	Bra023689	CHR17 (CHROMATIN REMODELING FACTOR17); ATP binding / DNA binding/ DNA-dependent ATPase/ helicase/ hydrolase, acting on acid anhydrides, in phosphorus-containing anhydrides/ nucleic acid binding/ nucleosome binding	10.49	0.62
PHD	Bra027316	PHD finger family protein	6.96	0

*FPKM* the expected number of Fragments *per* Kilobase of transcript sequence *per* Millions base pairs sequenced

## Discussion

In the diet of human beings, leafy vegetables always play an important role. Ozone pollution may cause serious injury to leafy vegetables and result in great losses of productivity and quality [16]. In our previous study, E-O_3_ caused visible injury and physiological alterations in Pak Choi but the molecular mechanism was not clear [19]. Previous reports showed that high O_3_ concentration triggers O_3_-responsive genes [20]. For examples, Ludwików et al. [9] reported the expression patterns of O_3_ stress-responsive Arabiodopsis genes with an emphasis on ROS-scavenging (e.g. catalase genes) and secondary metabolism (e.g. phenylpropanoid-related genes); Mahalingam et al. [21] classified O_3_-responsive genes into down-regulated (e.g. coding for proteins that function in chloroplast), early up-regulated (e.g. coding for membrane proteins and those involved in transcription and signaling), and late up-regulated genes (e.g. coding for membrane-associated or secretory proteins, and those involved in ROS-scavenging). In aspen trees, the genes related to defense and signaling were up-regulated while the genes in the carbohydrate metabolism were down-regulated [15]. Ludwików et al. [8] summarized the possible functions of differential expressed genes under O_3_ stress into several groups as follows: redox control, transcription, signal transduction, metabolism, and defense.

In this study, transcriptomic profiles of Pak Choi under NF or E-O_3_ were compared using RNA-Seq technology for the first time. It is noteworthy that the experiment was designed to simulate an acute O_3_ exposure according to realistic O_3_ concentrations in relatively clear area and in a highly O_3_-polluted area or a projected future [22, 23]. Based on the results of our previous physiological study with this species [19], we postulated that 2 days were sufficient to analyze the O_3_-responsive genes in Pak Choi. The current results not only characterized the essential key genes, but also unveiled potential metabolic pathways or TFs that are potentially involved in cellular regulation or adaptation against O_3_ stress. According to GO enrichment analysis, potential functions of DEGs under O_3_ stress have been summarized in several aspects, such as redox control, defense, transcription, signal transduction, and metabolism [8]. In this study, we found that the DEGs between E-O_3_ and NF were mainly related to defense, redox control and metabolism.

In particular, for the DEGs with increased expressions in plants under E-O_3_, the most enriched ones were ‘response to stimulus’, ‘oxidation-reduction process’, ‘response to stress’, ‘pathogenesis’ and ‘oxidoreductase activity’. The enrichment of genes which related to ‘pathogenesis’ indicated the similarity between plant response to O_3_ and pathogen infection, as both of these stresses triggered the production of ROS [24]. This result is consistent with the other reports in which pathogenesis-related (PR) proteins were induced by O_3_ [13, 25–27].

Ozone enters the leaves and subsequently triggers the production of ROS that leads to activation of ROS scavenging systems [6–8]. In our previous study, we showed that O_3_ treatment caused enhanced antioxidant enzyme activities in Pak Choi [19]. ROS are signaling molecules that often result in cell wall strengthening. In this study, cell wall-related genes were also found with increased expressions under O_3_ treatment. For example, xyloglucan endotransglycosylase (XETs) played key roles in repairing the damage of cell caused by O_3_ attack, promoting cell wall biogenesis and increasing cell or stomatal density. Our result showed that the expressions of XET coding genes increased in response to O_3_, which was consistent with previous reports [28–31], suggesting that cell wall modification is a common strategy for plant to adapt to high O_3_.

Previous reports showed that the expressions of some genes in ‘oxidation-reduction process’ or with ‘oxidoreductase activity’ increased in response to O_3_ stress in different plant species [4, 32–35], and similar results were observed with Pak Choi in this study. Proteomic analysis in poplar also showed that two enzymes with oxidoreductase activity increased under O_3_ exposure [36]. These activated oxidoreductase genes potentially function to avoid severe oxidative damage at the cellular level. Furthermore, KEGG pathway analysis also revealed significantly increased expressions of genes involved in the glutathione metabolism. Glutathione S-transferases (GSTs) have functions in preventing oxidative damage and keeping redox homeostasis [37–40]. Our results showed that the expression of DEGs encoding GSTs increased, suggesting that GSTs were involved in protecting the cell against O_3_ stress in this experiment. In addition, expressions of genes (*Bra025351* and *Bra039047*) homologous to *CYP81D11*, which putatively encodes cytochrome P450 monooxygenases, increased in the present study. It has been reported that expression of *CYP81D11* could be induced by osmotic stresses, ABA-, SA- and JA-treatments that the gene probably played important roles in plant detoxification processes [41, 42]. Furthermore, cytochrome P450 monooxygenases might be conjugated to glutathione or sugar moieties by either glutathione S-transferases or glycosyl transferases, and then the conjugates were transported to the apoplast or vacuole [43]. The higher expression of *GST* genes and the GO enrichment in ‘extracellular region’ or ‘apoplast’ probably revealed the detoxification processes of Pak Choi to O_3_ stress.

Oxidation of membrane lipid in the presence of ROS could lead to accumulation of omega-3 trienoic fatty acids (TFAs) that was the primary precursor of jasmonic acid (JA), one important phytohormone for defense [44–46]. As a feedback reaction, jasmonic acid (JA) acts to limit O_3_-lesion spread [7]. In the oxidative cell death cycle, jasmonates protect tissues from ROS-induced cell death and thus counteract the effects of salicylic acid and ethylene [7]. In the present study, the expression level of one putative JAZ7 encoding gene (*Bra021923*), which belonged to Tify family protein, was silenced when under O_3_ treatment. Yu et al. [47] reported that mutation of JAZ7 in the darkness might cause the up-regulation of the genes involved in sulphate metabolism, indole-glucosinolate biosynthesis, callose deposition, and JA-mediated signaling pathways. While the expression of gene (*Bra025713*) encoding JAZ1 protein, which acts to repress JA signaling, increased in this study [48, 49]. Our results of KEGG enrichment showed that the expressions of DEGs related to ‘sulfur metabolism’ and ‘glucosinolate biosynthesis’ increased, suggesting the JAZ7 and JAZ1 were both involved in O_3_ stress responses and might play opposite functions. This result indicated that O_3_ stress potentially activated JA-responsive genes and limited the spread of leaf cell death lesions to protect the healthy tissue, which was confirmed by the observation of foliar visible injury in our previous study [19].

In perceiving the O_3_ stress signals as well as JA signal, plants employ multifaceted signaling pathways to regulate their cellular responses. In this study, defense-related signaling kinase genes and transcription factor genes were also revealed in response to O_3_ stress. For example, the expression of a *SnRK2* family *serine/threonine-protein kinase* gene (*Bra015981*) increased [50]. The expressions of a number of defense-related *AP2/ERF*, *Trihelix*, *WRKY*, and *MYB* family genes also increased in response to O_3_ stress. It has been documented that members of the AP2/ERF family confer tolerance to multiple stresses [51, 52] and are key regulators of redox responsive gene networks [53]. In the present study, we also found that most of the DEGs encoding AP2/ERF family proteins had increased expression levels. Among them, *Bra024953* encoding *ERF5* had a higher expression level upon E-O_3_ than in NF. This result is also consistent with other previous studies [54]. In tomato, expression of *ERF5* was induced by abiotic stress, such as drought, wounding etc. [55]. It seems that the up regulation of *ERF5* is a common response to abiotic stresses. CBF4 is a regulator for adapting to drought stress [56]. Our results showed that this TF might also be involved in the response to O_3_ in Pak Choi.

Trihelix family genes play important roles in plant development, but their responses to abiotic stresses are indistinct to date [57]. Only a few Trihelix family genes were found responsive to abiotic stress such as cold or salt in *Brassica* species [57, 58]*.* For the first time, we found that the expressions of Trihelix family genes, *Bra005127*, *Bra005688*, and *Bra028899*, increased in Pak Choi when exposed to high O_3_ concentration. In particular, *Bra005688* encoded GT-3a ortholog, which has a function in binding GTTAC and is light inducible [59]. GT-3b, which is encoded by *Bra005127* can be induced by pathogen or salt stress [59, 60]. Our result suggested that these two TFs potentially played important roles in O_3_ stress regulation.

WRKY proteins with conserved WRKY motif and zinc finger-like domain function as transcriptional activators or repressors [61]. WRKY TFs have been found to be responsive to abiotic stresses such as O_3_ [62–64]. In this study, three DEGs encoding WRKY family proteins were identified and the expressions of them increased under E-O_3_ exposure. WRKY transcription factors were involved in the regulation of senescence-related processes [20, 62, 65]. It has been reported that 22 Pak Choi WRKY genes were differentially expressed in response to abiotic stresses, such as cold and salinity. [66]. In the present study, we found three new DEGs encoded WRKY28, WRKY48, and WRKY57, respectively, and were probably specifically related to O_3_ stress response.

The induction of MYBs also mediated transcriptional reprogramming in response to E-O_3_ [62, 64, 67]. MYBs could regulate the genes related to anthocyanin biosynthesis pathway and the JAZ-DELLA-MYBL2 module upstream of the MYB/bHLH/WD40 complex together mediated abiotic stress-caused anthocyanin accumulation in *Arabidopsis* [68]. Our results also showed that the expression of one gene encoding MYB family protein increased, which coincided with the accumulation of anthocyanin in O_3_-treated Pak Choi [19].

The other down-stream functional genes, besides those stated above (e.g. *XET*s & *GST*), also includes genes involved in sulfur metabolism and phenylpropanoid biosynthesis. Kimura et al. [69] reported that SnRK2.3, which enzyme’s putative encoding gene had increased expression, controls the production of O-acetyl-L-serine a putative signaling compound of the sulfur starvation response, and our KEGG enrichment result showed that the expression of DEG (*Bra038031*) encoded the enzyme catalyzing L-serine to O-acetyl-L-serine in the ‘sulfur metabolism’ pathway increased. These two genes (*Bra015981* and *Bra038031*) probably work together under the O_3_ caused sulfur starvation, among which *Bra038031* encoded the enzyme catalyzing L-serine to O-acetyl-L-serine and *Bra010645* encoded the enzyme catalyzing adenylyl sulfate to 3′-phosphoadenylyl sulfate.

The expressions of ten DEGs in ‘phenylpropanoid biosynthesis’ pathway increased. Proteins encoded by *Bra037007* and *Bra009105* were involved in catalyzing the alcohol to lignin. There were two DEGs which played a key role in catalyzing β-D-glucosyl-2-coumarinate into coumarinate. Flavonoid is a derivative product of the phenylpropanoid biosynthesis pathway. The *TRANSPARENT TESTA 12* (*TT12*) encodes a multidrug and toxic compound extrusion (MATE) vacuolar transporter that is required for flavonoid sequestration in developing seed coat of Arabidopsis [70]. Chai et al. [71] found that the *TT12* is less organ-specific and also expressed in leaves of Brassica. Our result showed that the gene (*Bra020862*) with increased expression and homologous to *AtTT12* in the leaves of Pak Choi was probably involved in the defense to O_3_ stress.

We also found that PR protein increased with the increasing expression of genes encoding GST. Plasmodesmata callose-binding protein 2 (PDCB2) is identified as a glycosylphosphatidylinositol (GPI)-anchor protein [72]. In the present study, the expression of DEG encoding PDCB2 increased under E-O_3_, suggesting that callose deposition might be enhanced, and cell to cell communication could be inhibited in O_3_-stressed Pak Choi [73].

The most enriched pathway of genes with decreased expression was ‘glycolysis/gluconeogenesis’. Some DEGs in this pathway were also involved in ‘carbon fixation in photosynthetic organisms’ and ‘pyruvate metabolism’ pathway. The lower biosynthesis of phosphoenolpyruvate from oxaloacetate, and D-Fructose 6-phosphate from D-Fructose 1, 6-bisphosphate were probably the main reasons for the impairment of photosynthesis and respiration under E-O_3._

## Conclusion

**Fig. 9:**
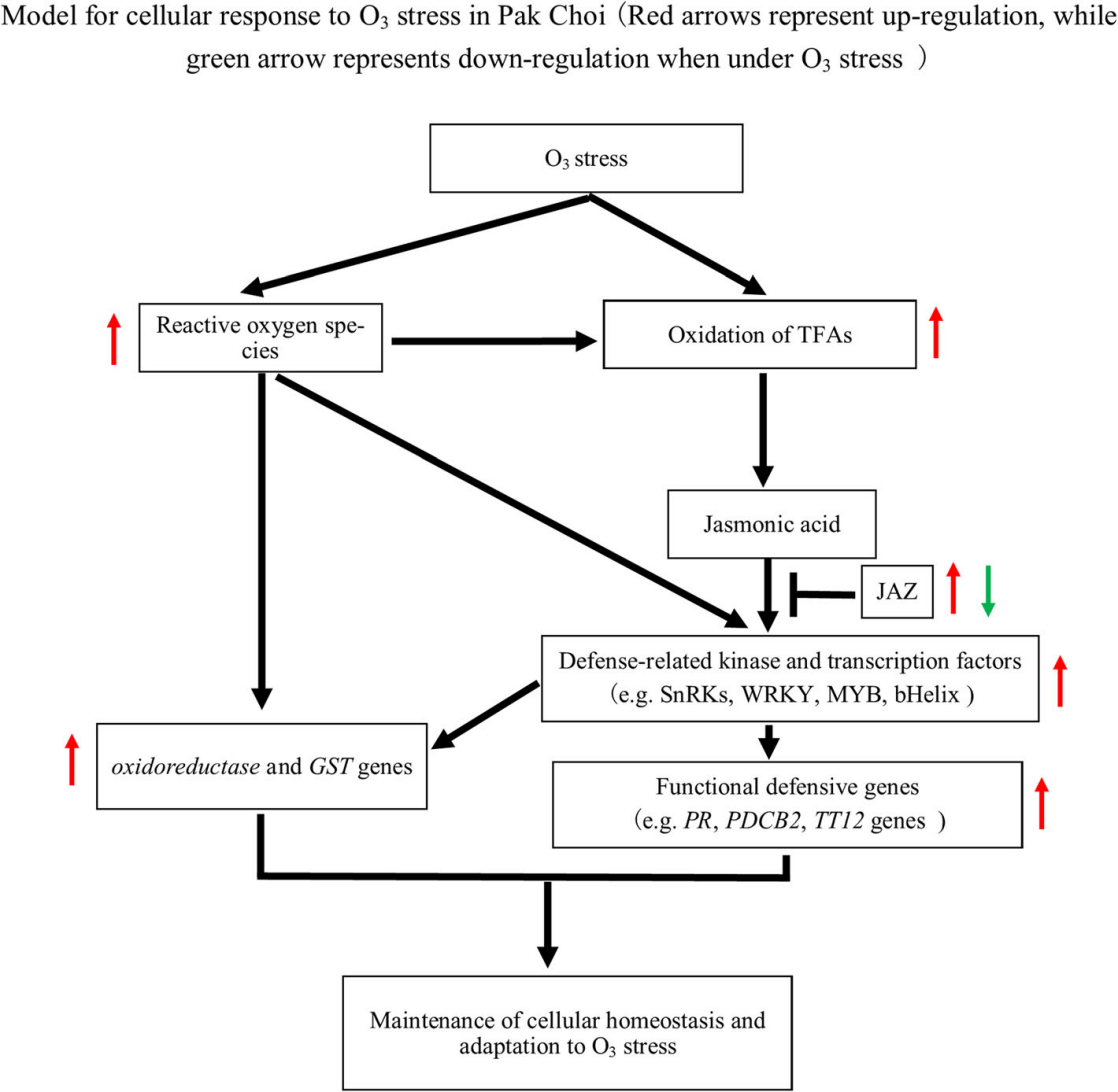
Proposed working model for acute O_3_–stress signaling network in Pak Choi

RNA-Seq was performed for Pak Choi plants exposed to NF and high O_3_ concentration. The transcriptomic comparison together with physiological analysis (published in [19]) revealed that O_3_ treatment led to ROS burst and then resulted in lipid oxidation and hormonal (esp. JA) signaling alterations. The signal transduction via phosphorylation (e.g. SnRK2 family kinases) ultimately affected the expression of defense-related transcription factors and down-stream stress-related functional genes (e.g. *PR* genes, *PDCB2* gene, sulfur metabolism and phenylpropanoid biosynthesis-related genes). The proposed working model for acute O_3_–stress signaling network was illustrated in Fig. 9. This result suggested that ROS metabolism, JA pathway and associated downstream functional genes worked together to maintain cellular homeostats and adaptation to O_3_ stress. The regulation of plant gene expression included multiple regulatory steps beyond transcriptional regulation; and the current result provided an overall, but not a complete, insight into O_3_ effect on Pak Choi. The specific DEGs coding for transcription factors, kinase and functional proteins could be valuable targets for genetic manipulation to improve the O_3_ stress tolerance of Pak Choi through experimental approaches in the future.

## Methods

### Plant material and treatments

One commonly cultivated variety of Pak Choi (‘Jingguan’) was selected in this study. Seeds were from Beijing Vegetable Research Center, Beijing Academy of Agriculture and Forestry Sciences. The seeds were sown in pots (10 cm in diameter), filled with a mixture of peat:vermiculite (3:1,v:v) at 25 °C in a greenhouse in July 2015. When they had five leaves in total, 20 seedlings were moved into open top chambers (OTCs) for O_3_ treatments.

The OTCs were located in Changping cropland area (40°19’N, 116°13′E), Beijing. Details on the OTCs are in Yuan et al. [74]. After 2 days’ adaption to OTC, 20 randomly-picked plants were moved into either of two OTCs. One OTC was fumigated with elevated O_3_ (E-O_3_) and the other one with non-filtered air (NF). Ozone was generated using pure oxygen by a generator and mixed with ambient air using a fan according to Hu et al. [75]. Ozone concentrations at approximately 10 cm above the plant canopy within the OTCs were continuously measured using an O_3_ analyzer (Model 49i, Thermo Scientific, USA). Plants were fumigated for 2 days (8 h *per* day, from 9:00–17:00) on 21st and 22nd August 2015, and the ozone metrics were calculated as AOT40 (the sum of the differences between hourly O_3_ concentrations and 40 ppb for each hour when the concentration is above 40 ppb during daylight hours) according to CLRTAP [76]. In NF, the 16 h mean O_3_ concentration was 30.79 ± 1.85 ppb, the maximum hourly O_3_ concentration was 42.60 ppb, and AOT40 was 0.004 ppm∙h (Additional file 5: Table S2). Under E-O_3_ exposure, the 16 h mean O_3_ concentration was 251.71 ± 8.15 ppb, the maximum hourly O_3_ concentration was 318.75 ppb, and AOT40 was 3.39 ppm∙h (Additional file 5: Table S2). At the end of the exposure, three biological replicates (plants) in each treatment were randomly selected for the following RNA-Seq analysis.

### RNA extraction and library preparation for transcriptome analysis

The second leaves from the top were sampled from three plants in each treatment, immediately frozen in liquid nitrogen for RNA preparation. Three μg RNAs *per* sample were used to generate sequencing libraries using the NEBNext® Ultra™RNA Library Prep Kit for Illumina® (NEB, USA). In brief, mRNA was isolated with poly-T oligo-attached magnetic beads, fragmented using NEBNext First Strand Synthesis Reaction Buffer (5X), and then reverse transcribed into first and second strand cDNA using random hexamer primer by M-MuLV Reverse Transcriptase (RNase H^−^) and DNA Polymerase I, respectively. The residual mRNA was removed by RNase H. The remaining overhangs were converted into blunt ends via exonuclease/polymerase activities, and adenylated at the 3′ ends with NEBNext Adaptor with a hairpin loop structure. Furthermore, the AMPure XP system (Beckman Coulter, Beverly, USA) was used to select 150~200 bp cDNA fragments. Then 3 μl USER Enzyme (NEB, USA) was used with adaptor-ligated, size-selected cDNA for 15 min at 37 °C followed by 5 min at 95 °C before PCR with Phusion High-Fidelity DNA polymerase, Universal PCR primers and Index (X) Primer. Finally, PCR products were purified in AMPure XP system and the library quality was assessed on the Agilent Bioanalyzer 2100 system. The obtained libraries were sequenced on an Illumina Hiseq 2500 platform to generate 125 bp paired-end reads.

### Analysis of Illumina sequencing results

In order to obtain clean data (clean reads), low quality reads, reads containing adapters, and reads containing poly-N of raw data were erased. After that Q20, Q30, GC-content of the clean data were calculated. Based on high quality clean data, the following analyses were conducted.

### Quantification of gene expression levels and differential expression analysis

The reference genome and gene model annotation files of *Brassica* were from its database website (http://brassicadb.org/brad/) [77]. Using TopHat (v2.0.12), clean data were mapped back onto the reference genome. The reads numbers mapped to each gene were calculated using the HTSeq (v0.6.1). The expected number of Fragments *per* Kilobase of transcript sequence *per* Millions base pairs sequenced (FPKM) of each gene was estimated based on reads numbers mapped to this gene and the gene length. Differentially expressed genes (DEGs) of plants under different treatments (three biological replicates *per* treatment) were identified by DESeq (*P* < 0.05, |log_2_(fold change)| > 0.8) [78]. Based on Wallenius non-central hyper-geometric distribution, Gene Ontology (GO) enrichment analysis (*P* < 0.05) of the DEGs was conducted by the GO seq R packages [79]. The statistical analysis (*P* < 0.05) of DEGs enrichment in Kyoto Encyclopedia of Genes and Genomes (KEGG) pathways was conducted using KOBAS 2.0 [80].

### Real-time quantitative RT-PCR (qRT-PCR) assay

Using qRT-PCR, the expression patterns of 12 genes (Gene ID: *Bra003517*, *Bra013923*, *Bra020878*, *Bra028899, Bra031485*, *Bra038089*, *Bra034061*, *Bra035732*, *Bra010802*, *Bra012938*, *Bra00944*, and *Bra025833*) were analyzed (Primers are listed in Additional file 6: Table S3). *Actin* was used as the reference gene according to [81]. In brief, cDNA was synthesized using ReverseTra Ace qPCR-RT Kit (Toyobo, Japan). The reverse transcription reaction system included 0.5 μL primer mix, 2 μL RNA template, 0.5 μL RT enzyme mix, 2 μL 5 × RT buffer, 5 μL ddH_2_O. In reference to the corresponding unigene sequence, gene-specific primers were designed using the online tool [GenScript Real-time PCR (TaqMan) Primer Design, https://www.genscript.com/ssl-bin/app/primer]. And the efficiency of the primer pairs were checked by serial dilutions of template cDNA as shown in Additional file 7: Figure S3. The cDNA was diluted to 100 ng μL^−1^ and then used for qRT-PCR test with each gene-specific primers and SYBR® Green Real time PCR Master Mix (Toyobo, Japan) on the Bio-Rad iQ5 real time system. Reactions were conducted at 96 °C for 1 min, 40 cycles of 95 °C for 15 s, 60 °C for 15 s and 72 °C for 45 s.

### Data analysis

qRT-PCR was conducted for each gene expression analysis using three biological replicates *per* treatment. The 16 h mean O_3_ concentration were calculated using 1 h average O_3_ concentration as replicate. Mean value and standard deviation were calculated in DPS based on Student’s t-test. The relative quantitation of gene expression of qRT-PCR was measured via the 2^−ΔΔCt^ method [82], with *actin* as the endogenous reference gene.

## Additional files

Additional file 1: Table S1. Summary of sequences analysis and RNA-Seq data. (DOCX 17 kb)

Additional file 2: Figure S1. The quality of raw reads of Pak Choi under elevated O_3_ (E-O_3_) and non-filtered air (NF) using RNA-Seq. (TIFF 1648 kb)

Additional file 3: Figure S2. The principle component analysis (PCA) of the reads of Pak Choi under elevated O_3_ (E-O_3_) and non-filtered air (NF) using RNA-Seq. (TIFF 80 kb)

Additional file 4: Differentially expressed genes (DEGs) in Pak Choi under two O_3_ exposure. (XLSX 61 kb)

Additional file 5: Table S2. Ozone concentrations in non-filtered air (NF) and elevated ozone (E-O_3_) exposure. (DOCX 15 kb)

Additional file 6: Table S3. The primers used for qRT -PCR. (DOC 33 kb)

Additional file 7: Figure S3. Effciency of primer pairs used in the qRT-PCR analysis. (TIFF 1374 kb)

## Abbreviations

bHLH: basic Helix-Loop-Helix
DEGs: Differentially expressed genes
E-O_3_: Elevated O_3_
FPKM: the expected number of Fragments *per* Kilobase of transcript sequence *per* Millions base pairs sequenced
GO: Gene Ontology
GSTs: Glutathione S-transferases
JA: Jasmonic acid
KEGG: Kyoto Encyclopedia of Genes and Genomes
NF: Non-filtered air
OTCs: Open top chambers
qRT-PCR: Real-time quantitative PCR
ROS: Reactive oxygen species

## References

[1] Hough AM, Derwent RG (1990). Changes in the global concentration of tropospheric ozone due to human activities. Nature.

[2] Ainsworth EA, Yendrek CR, Sitch S, Collins WJ, Emberson LD (2012). The effects of tropospheric ozone on net primary productivity and implications for climate change. Annu Rev Plant Biol.

[3] Feng Z, Sun J, Wan W, Hu E, Calatayud V (2014). Evidence of widespread ozone induced visible injury on plants in Beijing, China. Environ Pollut.

[4] Baier M, Kandlbinder A, Golldack D, Dietz KJ (2005). Oxidative stress and ozone: perception, signalling and response. Plant Cell Environ.

[5] Paoletti E (2007). Ozone impacts on forests. CAB Reviews: Perspectives in Agriculture, Veterinary Science, Nutrition and Natural Resources.

[6] Wohlgemuth H, Mittelstrass K, Kschieschan S, Bender J, Weigel HJ, Overmyer K, Kangasjärvi J, Sandermann H, Langebartels C (2002). Activation of an oxidative burst is a general feature of sensitive plants exposed to the air pollutant ozone. Plant Cell Environ.

[7] Kangasjärvi J, Jaspers P, Kollist H (2005). Signaling and cell death in ozone-exposed plants. Plant Cell Environ.

[8] Ludwików A, Sadowski J (2008). Gene networks in plant ozone stress response and tolerance. J Integr Plant Biol.

[9] Ludwików A, Gallois P, Sadowski J (2004). Ozone-induced oxidative stress response in Arabidopsis: transcription profiling by microarray approach. Cell Mol Biol Lett.

[10] Mahalingam R, Jambunathan N, Gunjan SK, Faustin E, Weng H, Ayoubi P (2006). Analysis of oxidative signaling induced by ozone in *Arabidopsis thaliana*. Plant Cell Environ.

[11] Xu E, Vaahtera L, Hõrak H, Hincha DK, Heyer AG, Brosché M (2015). Quantitative trait loci mapping and transcriptome analysis reveal candidate genes regulating the response to ozone in *Arabidopsis thaliana*. Plant Cell Environ.

[12] Puckette MC, Tang Y, Mahalingam R (2008). Transcriptomic changes induced by acute ozone in resistant and sensitive *Medicago truncatula* accessions. BMC Plant Biol.

[13] Lee S, Yun SC (2006). The ozone stress transcriptome of pepper (Capsicum Annuum L.). Mol Cell.

[14] Whaley A, Sheridan J, Safari S, Burton A, Burkey K, Schlueter J (2015). RNA-seq analysis reveals genetic response and tolerance mechanisms to ozone exposure in soybean. BMC Genomics.

[15] Gupta P, Duplessis S, White H, Karnosky DF, Martin F, Podila GK (2005). Gene expression patterns of trembling aspen trees following long-term exposure to interacting elevated CO_2_ and tropospheric O_3_. New Phytol.

[16] González-Fernández I, Elvira S, Calatayud V, Calvo E, Aparicio P, Sánchez M, Alonso R, Bermejo VB (2016). Ozone effects on the physiology and marketable biomass of leafy vegetables under Mediterranean conditions: spinach (Spinacia Oleracea L.) and Swiss chard (Beta Vulgaris L. Var. cycla). Agric Ecosyst Environ.

[17] Yoon HS, Lee H, Lee IA, Kim KY, Jo J (2004). Molecular cloning of the monodehydroascorbate reductase gene from *Brassica campestris* and analysis of its mRNA level in response to oxidative stress. Biochim Biophys Acta.

[18] Keck AS, Finley JW (2004). Cruciferous vegetables: cancer protective mechanisms of glucosinolate hydrolysis products and selenium. Integr Cancer Ther.

[19] Zhang L, Xiao S, Chen YJ, Xu H, Li YG, Zhang YW, Luan FS (2017). Ozone sensitivity of four Pakchoi cultivars with different leaf colors: physiological and biochemical mechanisms. Photosynthetica.

[20] Olbrich M, Betz G, Gerstner E, Langebartels C, Sandermann H, Ernst D (2005). Transcriptome analysis of ozone-responsive genes in leaves of European beech (*Fagus sylvatica* L.). Plant Biol.

[21] Mahalingam R, Shah N, Scrymgeour A, Fedoroff N (2005). Temporal evolution of the Arabidopsis oxidative stress response. Plant Mol Biol.

[22] Vingarzan R (2004). A review of surface ozone background levels and trends. Atmos Environ.

[23] Liu F, Zhu Y, Wang X (2008). Surface ozone pollution and its eco-environmental impacts in China ecol. Environment.

[24] Sharma YK, León J, Raskin I, Davis KR (1996). Ozone-induced responses in *Arabidopsis thaliana*: the role of salicylic acid in the accumulation of defense-related transcripts and induced resistance. Proc Natl Acad Sci U S A.

[25] Ernst D, Schraudner M, Langebartels C, JrH S (1992). Ozone-induced changes of mRNA levels of β-1, 3-glucanase, chitinase and ‘pathogenesis-related’ protein 1b in tobacco plants. Plant Mol Biol.

[26] Yalpani N, Enyedi AJ, León J, Raskin I (1994). Ultraviolet light and ozone stimulate accumulation of salicylic acid, pathogenesis-related proteins and virus resistance in tobacco. Planta.

[27] Eckey-Kaltenbach H, Kiefer E, Grosskopf E, Ernst D, JrH S (1997). Differential transcript induction of parsley pathogenesis-related proteins and of a small heat shock protein by ozone and heat shock. Plant Mol Biol.

[28] Gunthardt-Goerg MS, Matyssek R, Scheidegger C, Keller T (1993). Differentiation and structural decline in the leaves and bark of birch (*Betula pendula*) under low ozone concentrations. Trees.

[29] Paakkonen E, Metsarinne S, Holopainen T, Karenlampi L (1995). The ozone sensitivity of birch (*Betula pendula* Roth.) in relation to the developmental stage of leaves. New Phytol.

[30] Frey B, Scheidegger C, Gunthardt-Goerg MS, Matyssek R (1996). The effects of ozone and nutrient supply on stomatal response in birch (*Betula pendula*) leaves as determined by digital image-analysis and x-ray microanalysis. New Phytol.

[31] Kontunen-Soppela S, Riikonen J, Ruhanen H, Brosche M, Somervuo P, Peltonen P, Kangasjarvi J, Auvinen P, Paulin L, Keinanen M, Oksanen E, Vapaavuori E (2010). Differential gene expression in senescing leaves of two silver birch genotypes in response to elevated CO_2_ and tropospheric ozone. Plant Cell Environ.

[32] Rao MV, Paliyath G, Ormrod DP (1996). Ultraviolet-B-and ozone-induced biochemical changes in antioxidant enzymes of *Arabidopsis thaliana*. Plant Physiol.

[33] Sharma YK, Davis KR (1997). The effects of ozone on antioxidant responses in plants. Free Radic Biol Med.

[34] Agrawal GK, Rakwal R, Iwahashi H (2002). Isolation of novel rice (Oryza Sativa L.) multiple stress responsive MAP kinase gene, OsMSRMK2, whose mRNA accumulates rapidly in response to environmental cues. Biochem Biophys Res Commun.

[35] Paoletti E, Castagna A, Ederli L, Pasqualini S, Ranieri A, Manning WJ (2014). Gene expression in snap beans exposed to ozone and protected by ethylenediurea. Environ Pollut.

[36] Bohler S, Bagard M, Oufir M, Planchon S, Hoffmann L, Jolivet Y, Hausman JF, Dizengremel P, Renaut J (2007). A DIGE analysis of developing poplar leaves subjected to ozone reveals major changes in carbon metabolism. Proteomics.

[37] Agrawal GK, Jwa NS, Rakwal R (2002). A pathogen-induced novel rice (*Oryza sativa* L.) gene encodes a putative protein homologous to type II glutathione S-transferases. Plant Sci.

[38] Mueller LA, Goodman CD, Silady RA, Walbot V (2000). AN9, a petunia glutathione S-transferase required for anthocyanin sequestration, is a flavonoid-binding protein. Plant Physiol.

[39] Kampranis SC, Damianova R, Atallah M, Toby G, Kondi G, Tsichlis PN, Makris AM (2000). A novel plant glutathione S-transferase/peroxidase suppresses Bax lethality in yeast. J Biol Chem.

[40] Vijayakumar H, Thamilarasan SK, Shanmugam A, Natarajan S, Jung HJ, Park JI, Kim HR, Chung MY, Nou IS (2016). Glutathione transferases superfamily: cold-inducible expression of distinct GST genes in *Brassica oleracea*. Int J Mol Sci.

[41] Narusaka M, Seki M, Umezawa T, Ishida J, Nakajima M, Enju A, Shinozaki K (2004). Crosstalk in the responses to abiotic and biotic stresses in Arabidopsis: analysis of gene expression in *cytochrome P450* gene superfamily by cDNA microarray. Plant Mol Biol.

[42] Mueller S, Hilbert B, Dueckershoff K, Roitsch T, Krischke M, Mueller MJ, Berger S (2008). General detoxification and stress responses are mediated by oxidized lipids through TGA transcription factors in *Arabidopsis*. Plant Cell.

[43] Sandermann H (1992). Plant metabolism of xenobiotics. Trends Biochem Sci.

[44] Mène-Saffrané L, Dubugnon L, Chételat A, Stolz S, Gouhier-Darimont C, Farmer EE (2009). Nonenzymatic oxidation of trienoic fatty acids contributes to reactive oxygen species management in Arabidopsis. J Biol Chem.

[45] Rao MV, Koch JR, Davis KR (2000). Ozone: a tool for probing programmed cell death in plants. Plant Mol Biol.

[46] Tuominen H, Overmyer K, Keinänen M, Kollist H, Kangasjärvi J (2004). Mutual antagonism of ethylene and jasmonic acid regulates ozone-induced spreading cell death in Arabidopsis. Plant J.

[47] Yu J, Zhang Y, Di C, Zhang Q, Zhang K, Wang C, You Q, Yan H, Dai SY, Yuan JS, Xu W, Su Z (2016). JAZ7 negatively regulates dark-induced leaf senescence in Arabidopsis. J Exp Bot.

[48] Thines B, Katsir L, Melotto M, Niu Y, Mandaokar A, Liu G, Nomura K, He SY, Howe GA, Browse J (2007). JAZ repressor proteins are targets of the SCF^COI1^ complex during jasmonate signalling. Nature.

[49] Grunewald W, Vanholme B, Pauwels L, Plovie E, Inzé D, Gheysen G, Goossens A (2009). Expression of the Arabidopsis jasmonate signalling repressor JAZ1/TIFY10A is stimulated by auxin. EMBO Rep.

[50] Kulik A, Wawer I, Krzywińska E, Bucholc M, Dobrowolska G (2011). SnRK2 protein kinases—key regulators of plant response to abiotic stresses. Omics: J Integr Biol.

[51] Xu ZS, Chen M, Li LC, Ma YZ (2011). Functions and application of the AP2/ERF transcription factor family in crop improvement. J Integr Plant Biol.

[52] Mizoi J, Shinozaki K, Yamaguchi-Shinozakia K (1819). AP2/ERF family transcription factors in plant abiotic stress responses. Biochim Biophys Acta.

[53] Khandelwal A, Elvitigala T, Ghosh B, Quatrano RS (2008). Arabidopsis transcriptome reveals control circuits regulating redox homeostasis and the role of an AP2 transcription factor. Plant Physiol.

[54] Ludwików A, Kierzek D, Gallois P, Zeef L, Sadowski J (2009). Gene expression profiling of ozone-treated *Arabidopsis abi1td* insertional mutant: protein phosphatase 2C ABI1 modulates biosynthesis ratio of ABA and ethylene. Planta.

[55] Pan Y, Seymour GB, Lu C, Hu Z, Chen X, Chen G (2012). An ethylene response factor (*ERF5*) promoting adaptation to drought and salt tolerance in tomato. Plant Cell Rep.

[56] Haake V, Cook D, Riechmann J, Pineda O, Thomashow MF, Zhang JZ (2002). Transcription factor CBF4 is a regulator of drought adaptation in Arabidopsis. Plant Physiol.

[57] Luo J, Tang S, Mei F, Peng X, Li J, Li X, Yan X, Zeng X, Liu F, Wu Y, Wu G (2017). *BnSIP1-1*, a trihelix family gene, mediates abiotic stress tolerance and ABA signaling in *Brassica napus*. Front Plant Sci.

[58] Lee SC, Lim MH, Kim JA, Lee SI, Kim JS, Jin M, Kwon SJ, Mun JH, Kim HU, Hur Y, Park BS (2008). Transcriptome analysis in *Brassica rapa* under the abiotic stresses using *Brassica* 24K oligo microarray. Mol Cells.

[59] Ayadi M, Delaporte V, Li YF, Zhou DX (2004). Analysis of GT-3a identifies a distinct subgroup of trihelix DNA-binding transcription factors in Arabidopsis. FEBS Lett.

[60] Park HC, Kim ML, Kang YH, Jeon JM, Yoo JH, Kim MC, Park CY, Jeong JC, Moon BC, Lee JH, Yoon HW, Lee SH, Chung WS, Lim CO, Lee SY, Hong JC, Cho MJ (2004). Pathogen-and NaCl-induced expression of the SCaM-4 promoter is mediated in part by a GT-1 box that interacts with a GT-1-like transcription factor. Plant Physiol.

[61] Cho K, Shibato J, Agrawal GK, Jung YH, Kubo A, Jwa NS, Tamogami S, Satoh K, Higashi T, Kimura S, Saji H, Tanaka Y, Iwahashi H, Masuo Y, Rakwal R (2008). Integrated transcriptomics, proteomics, and metabolomics analyses to survey ozone responses in the leaves of rice seedling. J Proteome Res.

[62] Tosti N, Pasqualini S, Borgogni A, Ederli L, Falistocco E, Crispi S, Paolocci F (2006). Gene expression profiles of O_3_-treated Arabidopsis plants. Plant Cell Environ.

[63] Gao QM, Venugopal S, Navarre D, Kachroo A (2011). Low oleic acid-derived repression of jasmonic acid-inducible defense responses requires the WRKY50 and WRKY51 proteins. Plant Physiol.

[64] Iyer NJ, Tang Y, Mahalingham R (2013). Physiological, biochemical and molecular responses to a combination of drought and ozone in *Medicago truncatula*. Plant Cell Environ.

[65] Robatzek S, Somssich IE (2001). A new member of the Arabidopsis WRKY transcription factor family, AtWRKY6, is associated with both senescence- and defence-related processes. Plant J.

[66] Tang J, Wang F, Wang Z, Huang Z, Xiong A, Hou X (2013). Characterization and co-expression analysis of WRKY orthologs involved in responses to multiple abiotic stresses in Pak-choi (*Brassica campestris* Ssp. *chinensis*). BMC Plant Biol.

[67] Rizzo M, Bernardi R, Salvini M, Nali C, Lorenzini G, Durante M (2007). Identification of differentially expressed genes induced by ozone stress is sensitive and tolerant poplar hybrids. J Plant Physiol.

[68] Xie XB, Li S, Zhang RF, Zhao J, Chen YC, Zhao Q, Yao YX, You CX, Zhang XS, Hao YJ (2012). The bHLH transcription factor MdbHLH3 promotes anthocyanin accumulation and fruit colouration in response to low temperature in apples. Plant Cell Environ.

[69] Kimura T, Shibagaki N, Ohkama-Ohtsu N, Hayashi H, Yoneyama T, Davies JP, Fujiwara T (2006). Arabidopsis SNRK2.3 protein kinase is involved in the regulation of sulfur-responsive gene expression and O-acetyl-L-serine accumulation under limited sulfur supply. Soil Sci. Plant Nutr.

[70] Debeaujon I, Peeters AJM, Léon-Kloosterziel KM, Koornneef M (2001). The *TRANSPARENT TESTA 12* gene of Arabidopsis encodes a multidrug secondary transporter-like protein required for flavonoid sequestration in vacuoles of the seed coat endothelium. Plant Cell.

[71] Chai YR, Lei B, Huang HL, Li JN, Yin JM, Tang ZL, Wang R, Chen L (2009). *TRANSPARENTTESTA12* genes from *Brassica napus* and parental species: cloning, evolution, and differential involvement in yellow seed trait. Mol Gen Genomics.

[72] Elortza F, Nuhse TS, Foster LJ, Stensballe A, Peck SC, Jensen ON (2003). Proteomic analysis of glycosylphosphatidylinositol-anchored membrane proteins. Mol Cell Proteomics.

[73] Simpson C, Thomas C, Findlay K, Bayer E, Maule AJ (2009). An Arabidopsis GPI-anchor plasmodesmal neck protein with callose binding activity and potential to regulate cell-to-cell trafficking. Plant Cell.

[74] Yuan X, Calatayud V, Gao F, Fares S, Paoletti E, Tian Y, Feng Z (2016). Interaction of drought and ozone exposure on isoprene emission from extensively cultivated poplar. Plant Cell Environ.

[75] Hu EZ, Gao F, Xin Y, Jia HX, Li KH, Hu JJ, Feng Z (2015). Concentration- and flux-based ozone dose-response relationships for five poplar clones grown in North China. Environ Pollut.

[76] CLRTAP. Mapping Critical Levels for Vegetation, Chapter III of Manual on methodologies and criteria for modelling and mapping critical loads and levels and air pollution effects, risks and trends. UNECE Convention on Long-range Transboundary Air Pollution. 2015. http://www.icpmapping.org. Accessed 14 Apr 2016.

[77] Cheng F, Liu S, Wu J, Fang L, Sun S, Liu B, Li P, Hua W, Wang X (2011). BRAD, the genetics and genomics database for *Brassica* plants. BMC Plant Biol.

[78] Wang L, Feng Z, Wang X, Wang X, Zhang X (2010). DEGseq: an R package for identifying differentially expressed genes from RNA-Seq data. Bioinformatics.

[79] Young MD, Wakefield MJ, Smyth GK, Oshlack A (2010). Gene ontology analysis for RNA-seq: accounting for selection bias. Genome Biol.

[80] Mao X, Cai T, Olyarchuk JG, Wei L (2005). Automated genome annotation and pathway identification using the KEGG Orthology (KO) as a controlled vocabulary. Bioinformatics.

[81] Chandna R, Augustine R, Bisht NC (2012). Evaluation of candidate reference genes for gene expression normalization in *Brassica juncea* using real time quantitative RT-PCR. PLoS One.

[82] Livak KJ, Schmittgen TD (2001). Analysis of relative gene expression data using real-time quantitative PCR and the 2^−ΔΔCT^ method. Methods.

