# Supplementary material for: Transcriptomic analysis of Pak Choi under acute ozone exposure revealed regulatory mechanism against ozone stress
Source: BMC Plant Biol. 2017 Dec 8;17:236. doi: 10.1186/s12870-017-1202-4 (PMC5721698; doi:10.1186/s12870-017-1202-4)
Supplement: Supplementary file 1 — Summary of sequences analysis and RNA-Seq data. (DOCX 17 kb) [file 12870_2017_1202_MOESM1_ESM.docx]

**Table S1.** Summary of reads mapping of RNA-seq data.

| Sample name | E-O_3_-1 | E-O_3_-2 | E-O_3_-3 | NF-1 | NF-2 | NF-3 |
| --- | --- | --- | --- | --- | --- | --- |
| Clean reads | 50,266,492 | 55,061,140 | 52,966,538 | 56,549,076 | 51,426,790 | 57,313,470 |
| Clean bases | 7.54G | 8.26G | 7.94G | 8.48G | 7.71G | 8.6G |
| Error rate (%) | 0.01 | 0.01 | 0.01 | 0.01 | 0.01 | 0.01 |
| Q20 (%) | 98.12 | 98.05 | 97.97 | 97.86 | 98.05 | 98.01 |
| Q30 (%) | 95.18 | 95.08 | 94.86 | 94.68 | 95.13 | 95.03 |
| Total mapped (%) | 69.36 | 68.20 | 68.84 | 67.63 | 67.01 | 68.26 |
| Multiple mapped (%) | 1.42 | 1.48 | 1.46 | 1.31 | 1.16 | 1.53 |
| Uniquely mapped (%) | 67.93 | 66.72 | 67.37 | 66.33 | 65.85 | 66.73 |

Note: Q20 and Q30 represent percentages of bases with Phred values >20 or >30, respectively. The part of RNA-seq data of those grown in non-filtered air (NF, control) was published in Zhang et al. (2017) for the comparison of different Pak Choi varieties with contrasting anthocyanin contents.

Reference:

Zhang L., Xu B., Wu T., Yang Y., Fan L., Wen M., & Sui J. (2017). Transcriptomic profiling of two Pak Choi varieties with contrasting anthocyanin contents provides an insight into structural and regulatory genes in anthocyanin biosynthetic pathway. BMC genomics, 18(1), 288.
