# Supplementary figures and images for: Transcriptomic analysis of Pak Choi under acute ozone exposure revealed regulatory mechanism against ozone stress
Source: BMC Plant Biol. 2017 Dec 8;17:236. doi: 10.1186/s12870-017-1202-4 (PMC5721698; doi:10.1186/s12870-017-1202-4)

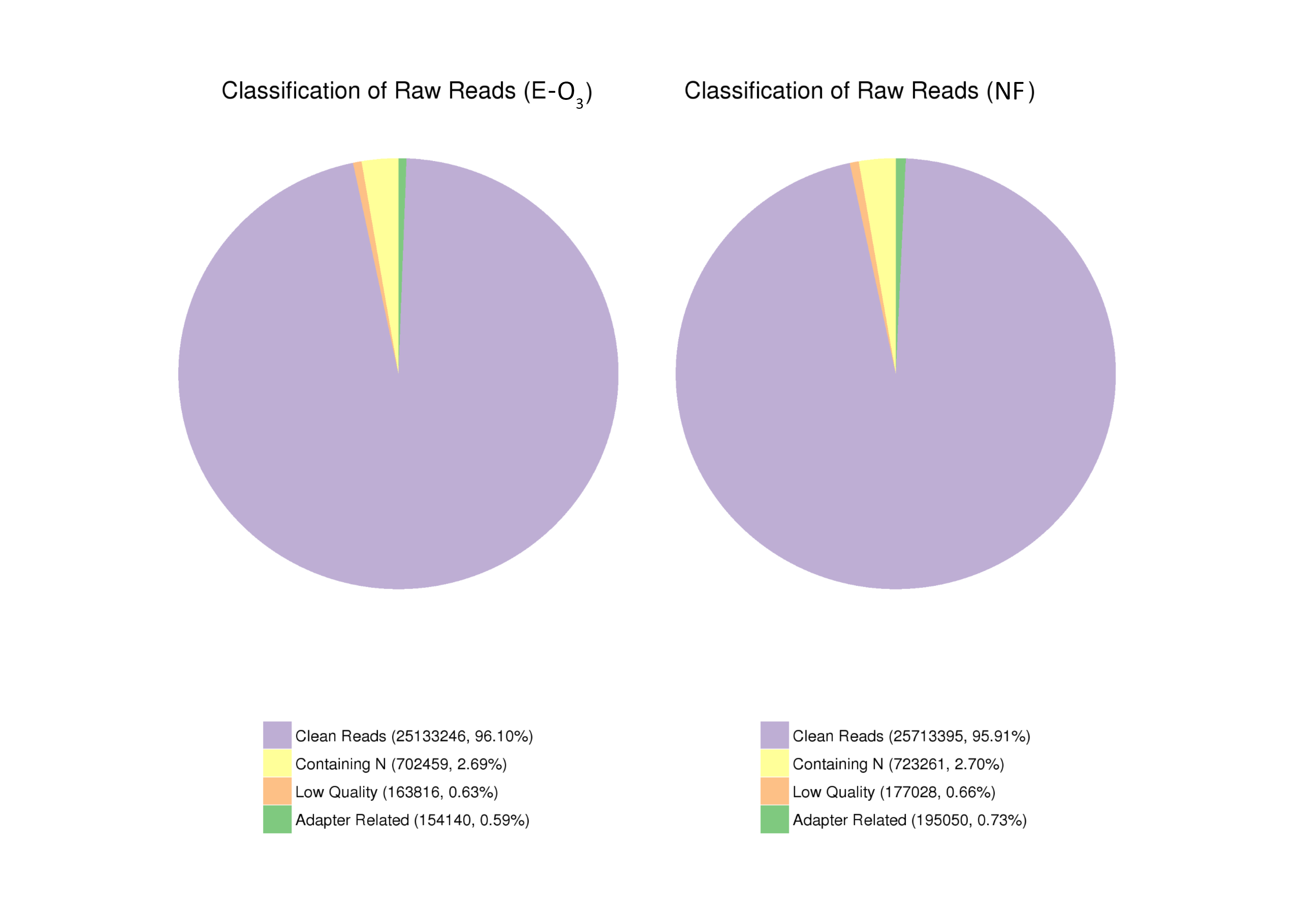

Supplement: Supplementary file 2 — The quality of raw reads of Pak Choi under elevated O3 (E-O3) and non-filtered air (NF) using RNA-Seq. (TIFF 1648 kb) [file 12870_2017_1202_MOESM2_ESM.tif]

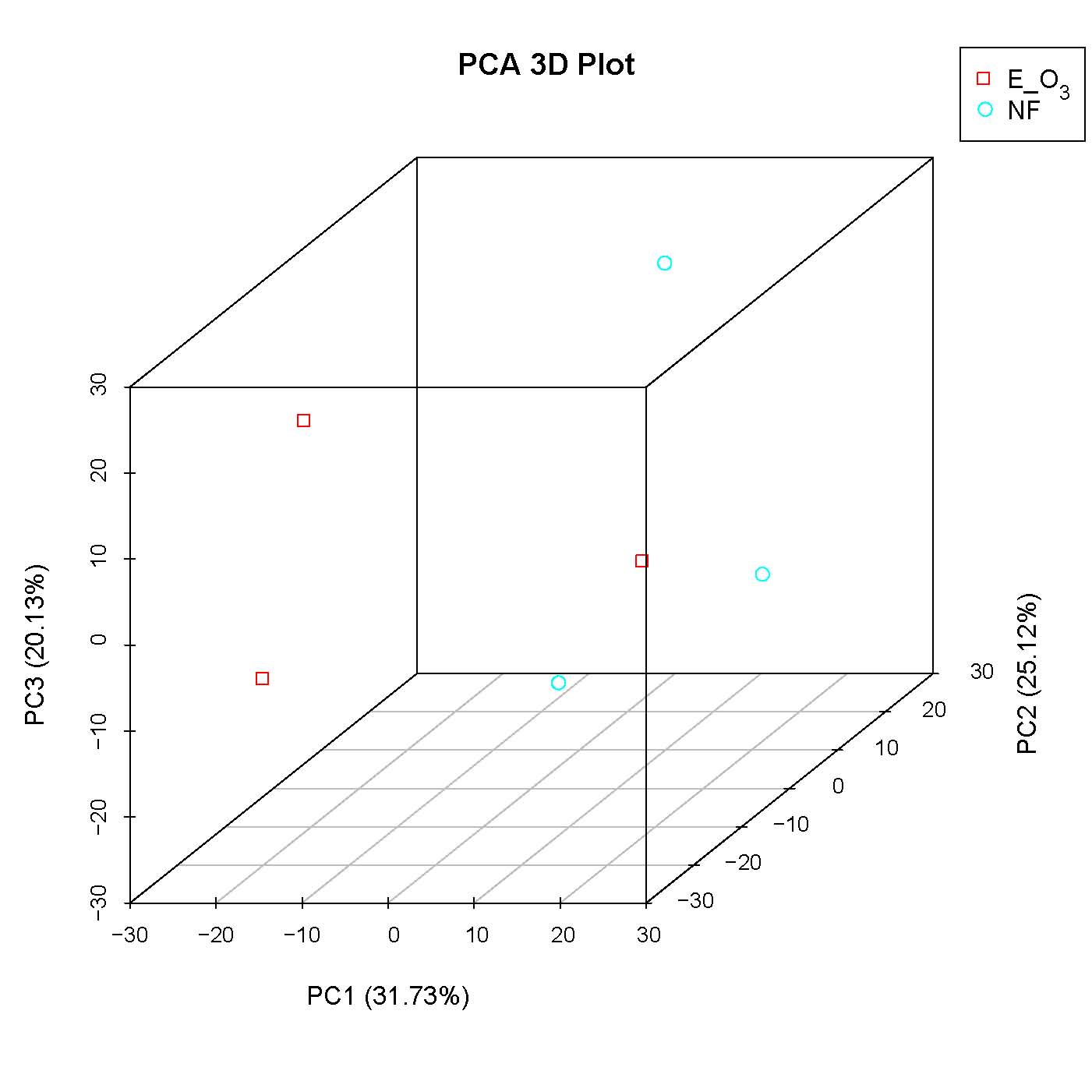

Supplement: Supplementary file 3 — The principle component analysis (PCA) of the reads of Pak Choi under elevated O3 (E-O3) and non-filtered air (NF) using RNA-Seq. (TIFF 80 kb) [file 12870_2017_1202_MOESM3_ESM.tif]

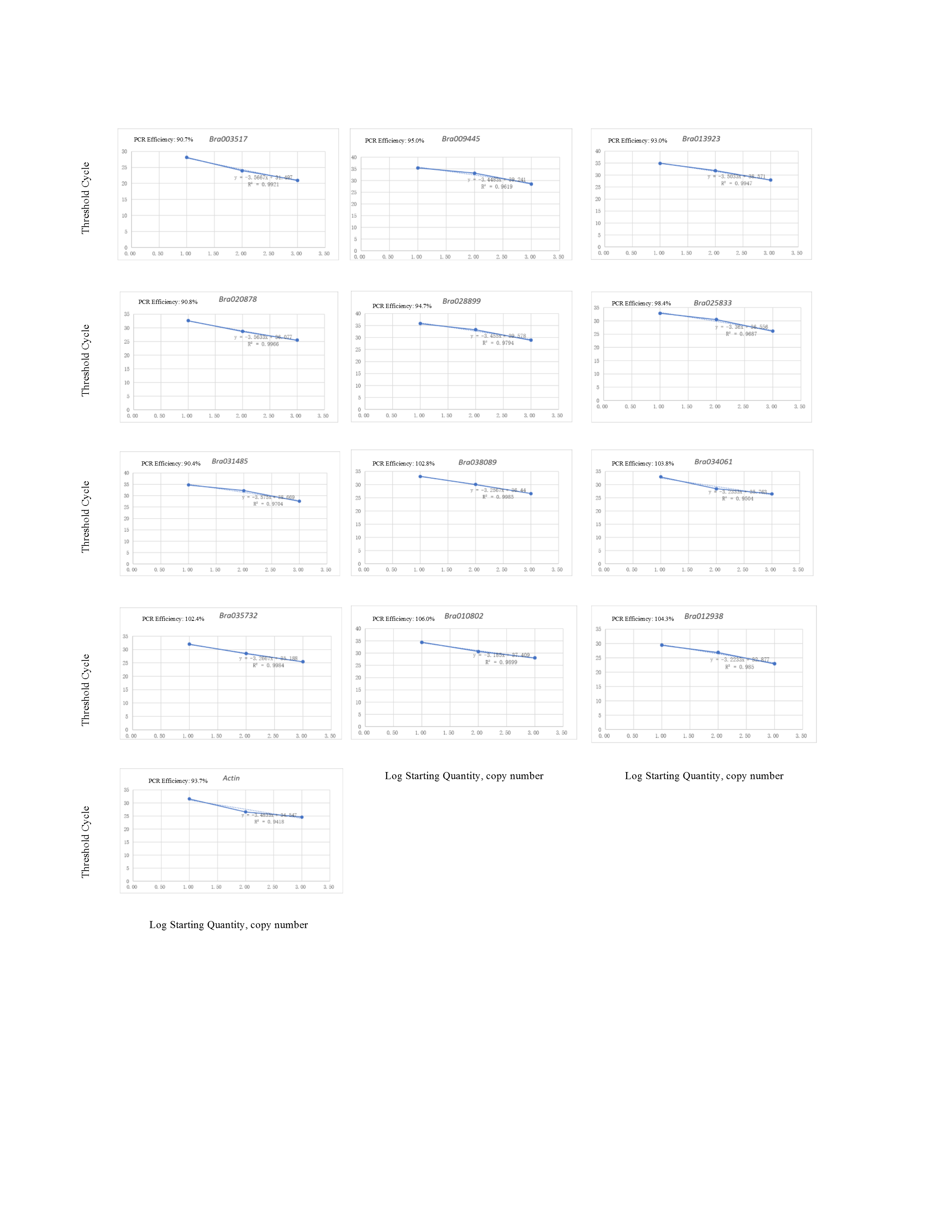

Supplement: Supplementary file 7 — Effciency of primer pairs used in the qRT-PCR analysis. (TIFF 1374 kb) [file 12870_2017_1202_MOESM7_ESM.tif]
