# Supplementary material for: Transcriptomic analysis of Pak Choi under acute ozone exposure revealed regulatory mechanism against ozone stress
Source: BMC Plant Biol. 2017 Dec 8;17:236. doi: 10.1186/s12870-017-1202-4 (PMC5721698; doi:10.1186/s12870-017-1202-4)
Supplement: Supplementary file 5 — Ozone concentrations in non-filtered air (NF) and elevated ozone (E-O3) exposure. (DOCX 15 kb) [file 12870_2017_1202_MOESM5_ESM.docx]

**Table S2.** Ozone concentrations in non-filtered air (NF) and elevated ozone (E-O_3_) exposure.

|  | NF | E-O_3_ |
| --- | --- | --- |
| 16h mean O_3_ concentration, ppb (Mean ± SD) | 30.79±7.40 | 251.71±32.59 |
| Maximum hourly O_3_ concentration (ppb) | 42.60 | 318.75 |
| AOT40 (ppm^.^h) | 0.004 | 3.39 |
