# Supplementary material for: Transcriptomic analysis of Pak Choi under acute ozone exposure revealed regulatory mechanism against ozone stress
Source: BMC Plant Biol. 2017 Dec 8;17:236. doi: 10.1186/s12870-017-1202-4 (PMC5721698; doi:10.1186/s12870-017-1202-4)
Supplement: Supplementary file 6 — The primers used for qRT -PCR. (DOC 33 kb) [file 12870_2017_1202_MOESM6_ESM.doc]

**Table S3.** The primers used for qRT-PCR.

| Gene | Forward primer (5’-3’) | Reverse primer (5’-3’) |
| --- | --- | --- |
| Actin | GTTGCTATCCAGGCTGTTCT | AGCGTGAGGAAGAGCATAAC |
| Bra003517 | AGTGGTAAGCTGGTGGGTTC | GATCAAGGGAACGAGAGAGC |
| Bra013923 | GCATGTAGCAGAACCTCGAA | AGAGATGTCCACATCCACGA |
| Bra020878 | GCTACAAGTGAGCGTGGAGA | TGGATGAAGATCCTGTGGAA |
| Bra028899 | GAAACCGCGGGTAGTACATT | CCTTCTCTCTCGCCTTCATC |
| Bra025833 | TTTGTTGATGCACTTGCTGA | TGTCCAAGAGATTCGTCTGC |
| Bra031485 | CCACAGGGTCTACCGAGATT | CAGACATCATCTCCCTGGTG |
| Bra038089 | GAACCGGAGACTTGCTCAAT | CTGCGTCAAGACCCACTAAA |
| Bra034061 | CATATTGGTTTGGAATTGCG | CCATGAGCTTCTCCTTAGGC |
| Bra035732 | ACCTCCTTACCGATTTCCCT | TAGGAACTGAAATGGTGGCA |
| Bra010802 | GAGATGGTGACGGAAGGATT | CCATCTTCTCCACCTCTTCC |
| Bra012938 | GTGATACCACGGAGACAACG | ACGGCGTTAAAGGTACTGCT |
| Bra009445 | TCGAGAGAACTCGTTTGCAC | TACCGCAATACCAATGAGGA |
